# Targeting amino acid-metabolizing enzymes for cancer immunotherapy

**DOI:** 10.3389/fimmu.2024.1440269

**Published:** 2024-08-14

**Authors:** Yvonne Grobben

**Affiliations:** Oncolines B.V., Oss, Netherlands

**Keywords:** cancer immunotherapy, immunosuppression, amino acid metabolism, tryptophan, arginine, glutamine, IDO1, IL4I1

## Abstract

Despite the immune system’s role in the detection and eradication of abnormal cells, cancer cells often evade elimination by exploitation of various immune escape mechanisms. Among these mechanisms is the ability of cancer cells to upregulate amino acid-metabolizing enzymes, or to induce these enzymes in tumor-infiltrating immunosuppressive cells. Amino acids are fundamental cellular nutrients required for a variety of physiological processes, and their inadequacy can severely impact immune cell function. Amino acid-derived metabolites can additionally dampen the anti-tumor immune response by means of their immunosuppressive activities, whilst some can also promote tumor growth directly. Based on their evident role in tumor immune escape, the amino acid-metabolizing enzymes glutaminase 1 (GLS1), arginase 1 (ARG1), inducible nitric oxide synthase (iNOS), indoleamine 2,3-dioxygenase 1 (IDO1), tryptophan 2,3-dioxygenase (TDO) and interleukin 4 induced 1 (IL4I1) each serve as a promising target for immunotherapeutic intervention. This review summarizes and discusses the involvement of these enzymes in cancer, their effect on the anti-tumor immune response and the recent progress made in the preclinical and clinical evaluation of inhibitors targeting these enzymes.

## 1 Introduction

Cancer arises from the accumulation of genetic and epigenetic alterations, conferring selective growth advantage to transformed cells (1, 2). Associated with these alterations is generally a diverse set of tumor-expressed antigens, including aberrantly expressed self-antigens and neoantigens resulting from somatic mutations (3). Although this antigenic diversity provides the immune system with ample opportunity to recognize and destroy cancerous cells, an effective anti-tumor immune response is absent in many human cancers (4). Mechanisms facilitating the immune escape of tumor cells include the downregulation or loss of tumor antigens or antigen-presenting machinery, the impairment of T-cell trafficking and infiltration into tumors, and the induction of immunosuppressive factors and cells in the tumor microenvironment (TME) (5, 6).

Over the recent decades, cancer immunotherapy has emerged as a revolutionary approach to reinvigorate host anti-tumor immunity (7). By alleviating negative regulation of T-cell activation, antibodies targeting inhibitory immune checkpoint proteins, including programmed death 1 (PD-1), its ligand PD-L1, and cytotoxic T-lymphocyte-associated protein 4 (CTLA-4), have produced durable clinical responses in a subset of cancer patients (8–10). Other immunotherapeutic treatment modalities, such as cytokine therapy, cancer vaccines and adoptive cell transfer, have additionally been developed to amplify pre-existing immune reactivity in patients, or generate new tumor-specific immune responses (7, 11). However, while cancer immunotherapies represent attractive alternatives to conventional and targeted therapies in terms of efficacy and tolerability, many patients experience primary or acquired resistance (12, 13), necessitating the development of alternative strategies or combinatorial therapies.

A critical hurdle for successful immunotherapeutic treatment of cancer patients is the complex and heterogeneous nature of the TME (14). Within this environment, tumor-induced accumulation of immunosuppressive cells, such as regulatory T cells and myeloid-derived suppressor cells (MDSCs), can promote profound tolerance to cancerous cells (15, 16). Molecular mechanisms employed by these suppressive populations as well as by tumor cells themselves include the expression of inhibitory receptors or their ligands (8), and the secretion of immunosuppressive cytokines (17). In addition, upregulation of metabolic enzymes by any of these participants can deprive the TME of nutrients essential to proliferating T cells, or expose them to high levels of immunosuppressive metabolites (18). Finally, the frequently hypoxic and acidic conditions surrounding tumor-infiltrating T cells can further attenuate their function (19).

In this review, the role of a specific group of metabolic enzymes—*i.e.*, those metabolizing amino acids—in the escape of tumor cells from immune surveillance will be summarized; advances in the therapeutic targeting of these enzymes will be highlighted; and current challenges and opportunities in this field will be discussed.

## 2 Amino acid-metabolizing enzymes involved in tumor immune escape

Amino acids are integral to cellular homeostasis and proliferation, serving as precursors for protein synthesis and constituting key metabolic intermediates in energy production and various biosynthetic pathways. In naïve T cells, only minimal uptake of amino acids is required to maintain homeostasis, which is attributable to the metabolically quiescent state of these cells (20). However, upon cognate antigen engagement and co-stimulation, T cells drastically alter their metabolism to meet the energetic and anabolic needs of rapid growth and proliferation (21). Being auxotrophic for most amino acids (22), this requires activated T cells to strongly increase both essential and non-essential amino acid uptake (23). In the case of tumor-infiltrating T cells, this has to be achieved amidst the highly competitive and dynamic settings of the TME.

**Figure 1:**
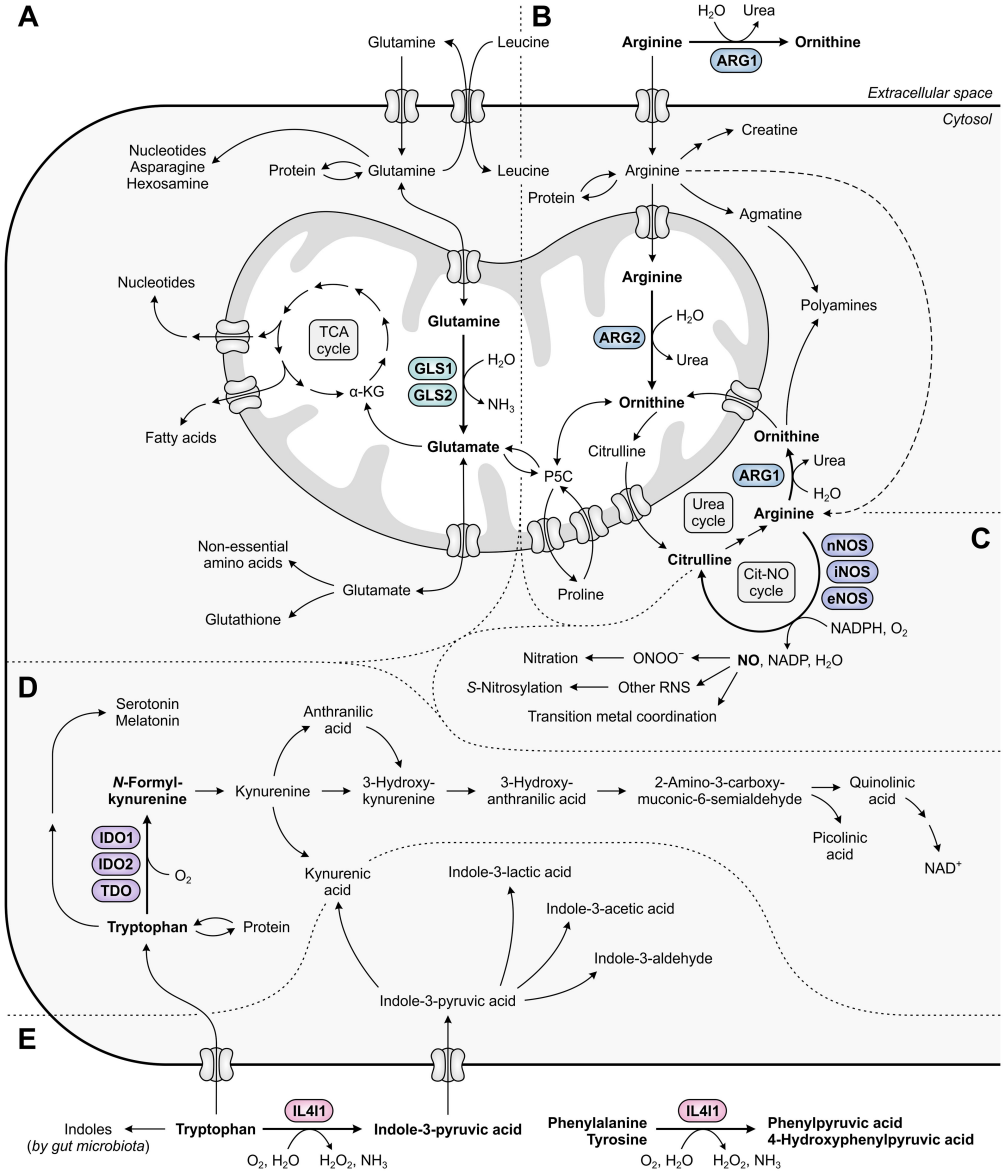
Metabolic fates of amino acids and metabolites involved in tumor immunosuppression, with relevant amino acid-metabolizing enzymes indicated. **(A)** Glutamine that has entered the cell is incorporated into proteins, contributes to the biosynthesis of nucleotides, asparagine and hexosamine, and is imported into mitochondria. Within mitochondria, GLS1 and GLS2 convert glutamine into glutamate, which contributes to the generation of tricarboxylic acid (TCA) cycle intermediates and derivatives, and to the cytosolic biosynthesis of non-essential amino acids and glutathione. **(B)** Arginine can be metabolized to ornithine either extracellularly or cytosolically (as the final step of the urea cycle) by ARG1, or in the mitochondria by ARG2. Ornithine can subsequently be used for a new cycle of ammonia detoxification, or can be converted into polyamines, proline or glutamate. Alternative fates of arginine include incorporation into proteins and production of creatine, polyamines and nitric oxide (NO). **(C)** NO is produced from arginine by nNOS, iNOS or eNOS, and can be converted into different reactive nitrogen species (RNS) that can alter the structure and function of various biomolecules through nitration, *S*-nitrosylation or transition metal coordination. **(D)** Tryptophan serves as a fundamental protein building block, and can be metabolized along the serotonin and kynurenine pathways to generate a variety of bioactive metabolites. **(E)** Tryptophan can additionally be metabolized into indoles by both host cell-secreted IL4I1 and gut microbiota, of which the former also metabolizes phenylalanine and tyrosine. Other metabolic pathways of phenylalanine and tyrosine are not shown in this figure.

Within the tumor landscape, metabolic reprogramming is not a unique feature of activated T cells, as highly proliferative cancer cells have similar metabolic requirements. Cancer cells often avidly consume energetic nutrients, particularly glucose and glutamine (24, 25), causing them to outcompete T cells for these respiratory fuels and biosynthetic precursors. This voracious phenotype is governed by the upregulation of transporter proteins as well as metabolic enzymes, including the glutamine-metabolizing enzyme glutaminase 1 (GLS1) (**Figure 1A**) (26, 27). Expression of other amino acid-metabolizing enzymes can additionally be exploited by either tumor or infiltrating immunosuppressive cells. These enzymes include arginase 1 (ARG1) (28, 29), inducible nitric oxide synthase (iNOS) (30, 31), indoleamine 2,3-dioxygenase 1 (IDO1) (32–34), tryptophan 2,3-dioxygenase (TDO) (35, 36) and interleukin 4 induced 1 (IL4I1) (**Figures 1B–E**) (37). Not only may the activities of these enzymes directly serve to potentiate tumor malignant properties through different mechanisms, it is their ability to promote tumor growth through suppression of immune responses that is their main common denominator (28, 33, 36–39).

**Figure 2:**
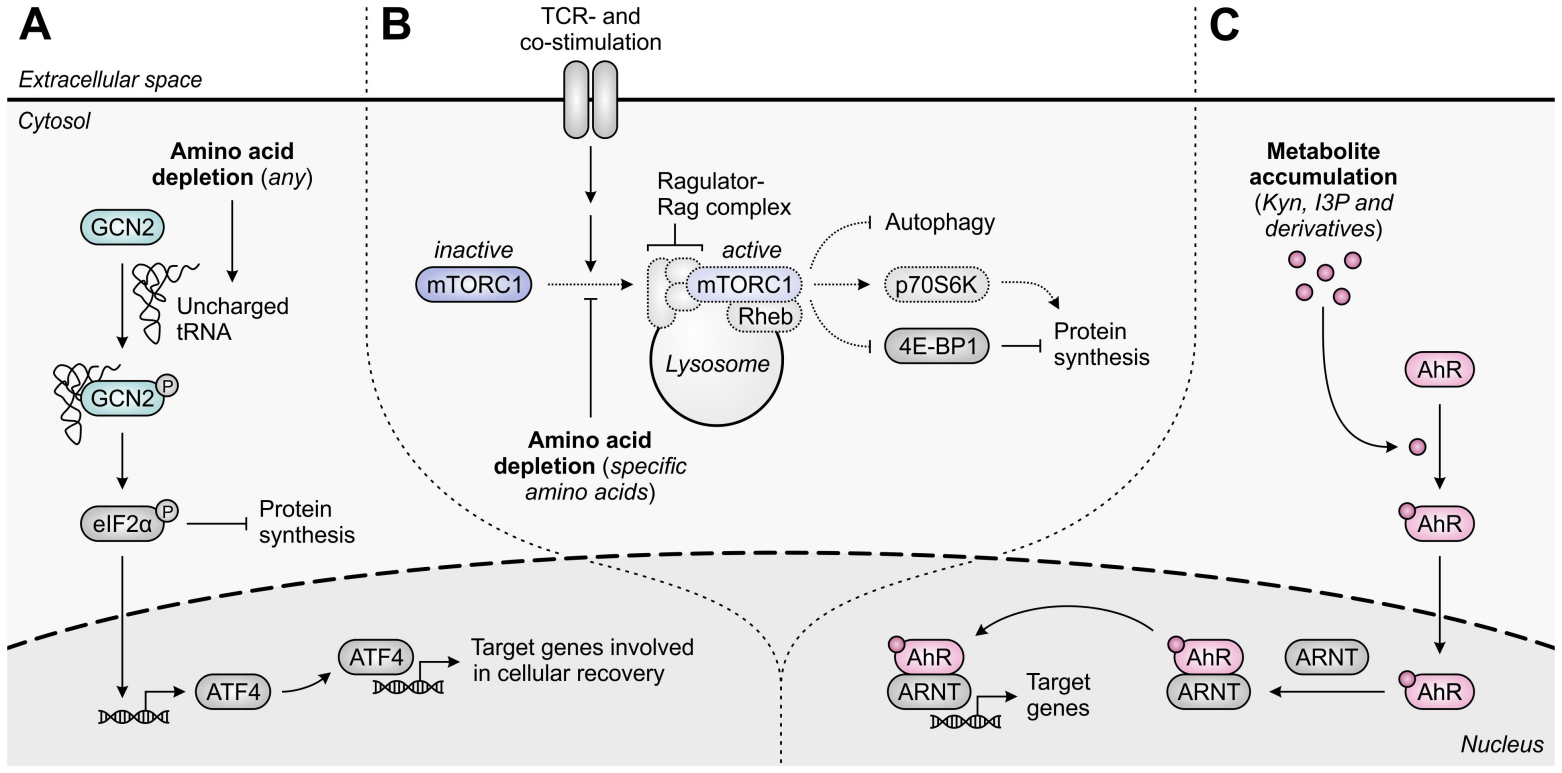
Molecular pathways underlying immunosuppression in T cells upon amino acid depletion or metabolite accumulation. **(A)** The general control nonderepressible 2 (GCN2) kinase is activated by uncharged tRNA, which accumulates in cells upon depletion of any amino acid. Activated GCN2 phosphorylates eIF2α, which halts global protein synthesis and induces ATF4 expression, which in turn induces the transcription of ATF4 target genes that promote cellular recovery. **(B)** The mammalian target of rapamycin complex 1 (mTORC1) is recruited to the lysosomal surface upon activation of the Ragulator-Rag complex by specific amino acids, including arginine and leucine, and is subsequently activated by T-cell receptor (TCR)- and co-stimulatory signal-activated Rheb. Activated mTORC1 promotes protein synthesis through regulation of p70S6K and 4E-BP1 activity, and inhibits autophagy. Amino acid depletion impedes these processes, as indicated by the dotted outlines and arrows. **(C)** The aryl hydrocarbon receptor (AhR) is translocated to the nucleus upon binding of an agonist such as tryptophan-derived kynurenine (Kyn), indole-3-pyruvic acid (I3P) or their downstream metabolites. In the nucleus, the AhR binds to the AhR nuclear translocator (ARNT) and induces the transcription of its target genes, which are involved in a variety of physiological processes.

A common mechanism of immunosuppression exerted by different amino acid-metabolizing enzymes relies on the amino acid dependency of activated T cells. As in all eukaryotic cells, the intracellular availability of amino acids in T cells is continuously monitored through at least two distinct pathways, involving either the general control nonderepressible 2 (GCN2) kinase (40) or the mammalian target of rapamycin complex 1 (mTORC1) (41). Activation of the GCN2 pathway occurs upon accumulation of uncharged tRNAs consequent to amino acid withdrawal (**Figure 2A**) (42), whereas T-cell receptor (TCR)-induced mTORC1 signaling is inhibited upon insufficiency of selected amino acids (**Figure 2B**) (41). Through independent mechanisms, either perturbation induces a global reduction in translation initiation. Moreover, GCN2 activation results in the selective induction of genes aiding in cellular recovery, while mTORC1 inhibition promotes autophagy (**Figures 2A, B**) (40, 43). As a consequence, amino acid deprivation can severely impact T cell functionality.

A second suppressive mechanism shared by distinct amino acid-metabolizing enzymes is the accumulation of immunosuppressive metabolites within the TME. Among the different metabolites generated by these enzymes, activation of the aryl hydrocarbon receptor (AhR) is the most represented mechanism of immunosuppression (**Figure 2C**) (36, 37, 44). The AhR is a ligand-activated transcription factor expressed by most human cell types, including various cells of the immune system, in which it regulates the transcription of numerous target genes. Through sensing of a broad range of exogenous and endogenous ligands, including tryptophan-derived metabolites, the AhR controls various physiological processes, including cell cycle progression, cellular motility and immune cell function (45, 46).

In the following sections, each of the amino acid-metabolizing enzymes involved in tumor immune escape will be discussed separately, as this allows for the depth of review to be adequately coordinated with the extent of knowledge available. As an exception, IDO1 and TDO will be discussed jointly, as these enzymes demonstrate highly overlapping mechanisms of action.

## 3 The glutamine-metabolizing enzyme GLS1

**Figure 3:**
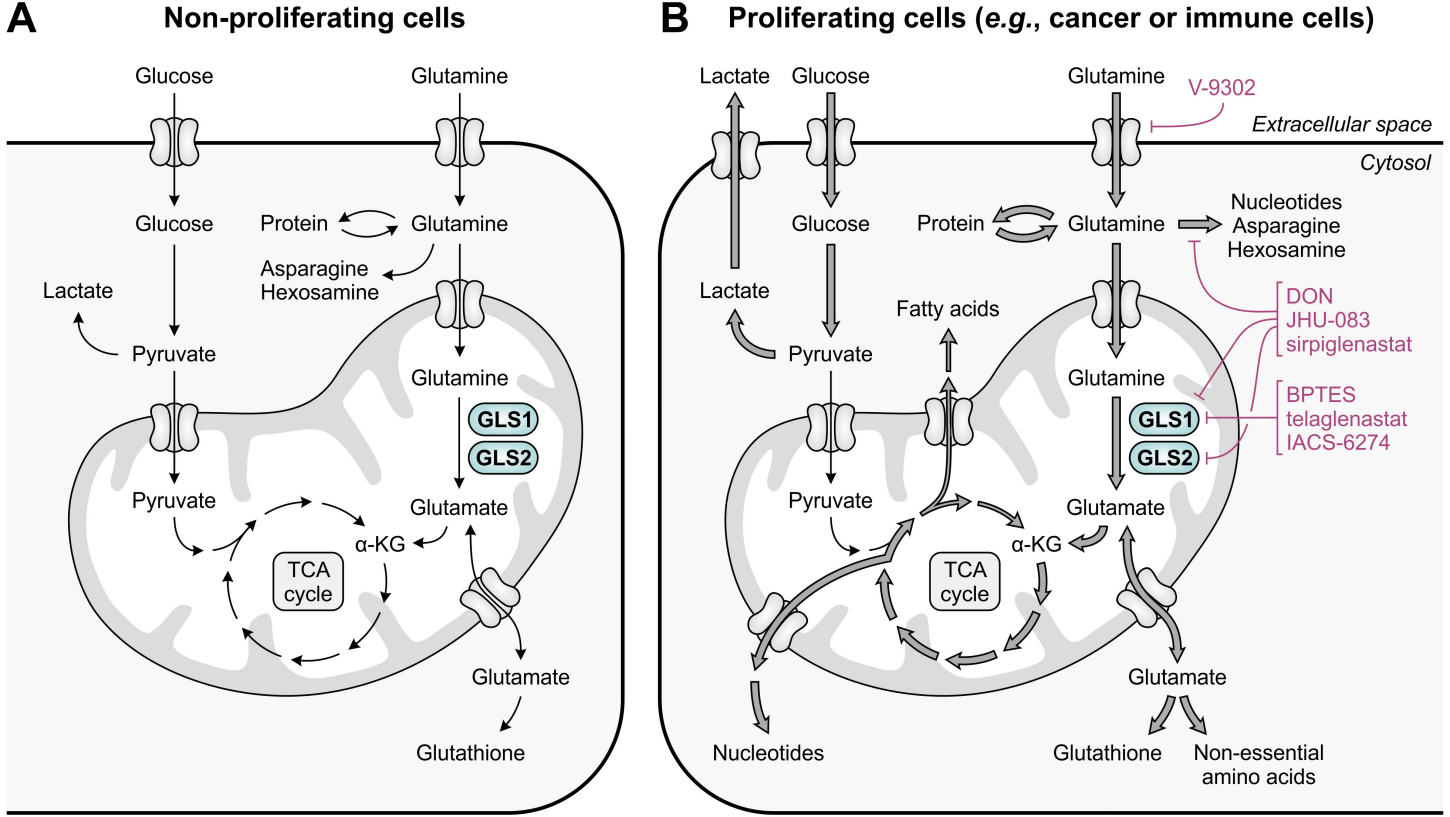
Glutamine and glucose metabolism in non-proliferating and proliferating cells. **(A)** In non-proliferating cells, glucose is primarily responsible for energy generation through glycolysis, the tricarboxylic acid (TCA) cycle and oxidative phosphorylation, whereas glutamine contributes to a number of biosynthetic processes. **(B)** In proliferating cells, enhanced glucose import facilitates the production of energy and glycolytic intermediates through aerobic glycolysis, which diverts pyruvate away from the TCA cycle. To meet the high demand for TCA cycle intermediates and derivatives, glutamine import and glutaminolysis are considerably enhanced, which concurrently facilitates the upregulation of biosynthetic pathways requiring glutamine or glutamate as a substrate. The excessive import or metabolism of glutamine by proliferating cells can be restricted through use of selective GLS1 inhibitors, broad-spectrum inhibitors of glutamine-utilizing enzymes, or glutamine uptake inhibitors, of which relevant examples are provided in the figure.

Glutamine is the most abundant free amino acid in humans, both in circulation and in the intracellular environment (47). It is considered conditionally essential, as it can be adequately obtained from *de novo* synthesis and protein turnover in healthy individuals, but may become insufficient during critical illness or injury (48). Aside from its key role in protein synthesis, glutamine contributes to numerous biosynthetic pathways, including those directed towards synthesis of nucleotides, non-essential amino acids, fatty acids and glutathione (**Figure 1A**) (49). Moreover, glutamine can serve as a significant source of energy in cells with high energetic demands (50). In non-proliferating cells, this role is primarily reserved for glucose, which is efficiently used in glycolysis, the tricarboxylic acid (TCA) cycle and oxidative phosphorylation for generation of ATP and biosynthetic precursors (**Figure 3A**) (51). In contrast, energy production in most cancer cells is shifted towards the inefficient use of glucose in aerobic glycolysis (“the Warburg effect”), achieved through re-programming of metabolic pathways (52). This altered metabolism yields important glycolytic intermediates required for various anabolic processes, but concurrently restricts entry of glucose into the TCA cycle. To compensate for this metabolic shift, cancer cells often become addicted to exogenous glutamine, which can replenish TCA cycle intermediates through its downstream metabolite α-ketoglutarate in a process called glutaminolysis (**Figure 3B**) (51).

Glutamine can be transported into cells through many different transporters of the solute carrier (SLC) family (53). Conversion of glutamine into α-ketoglutarate is subsequently initiated by glutaminase (GLS) enzymes, which catalyze the deamidation of glutamine to glutamate and act as the rate-limiting enzymes for glutamine entry into the TCA cycle. In mammalian cells, GLS is encoded by two genes, *GLS1* (or kidney-type glutaminase; KGA) and *GLS2* (or liver-type glutaminase; LGA). Expression of *GLS1* occurs ubiquitously across human extrahepatic tissues (54, 55), and is regulated by the c-Myc oncoprotein, which coordinately controls cellular glutamine uptake (26, 27). Elevated tumoral GLS1 expression is found across a variety of cancer types, and is frequently correlated with poor patient prognosis (56–60). In contrast, *GLS2* is identified as a target gene of the tumor suppressor protein p53 (61, 62), with expression restricted primarily to the liver, pancreas, brain and pituitary gland (54, 55). Along with its decreased expression in various cancer types (59, 63), GLS2 is therefore generally regarded to have a tumor-suppressive function. This may be related to its role in the promotion of an iron-dependent form of cell death termed ferroptosis (64), or its ability to act as a binding protein independent of its glutaminase activity (65). In contrast, expression of GLS2 can also be promoted by the oncogenic n-Myc protein (66), and upregulated *GLS2* expression in breast cancer tissues is found to be correlated with poor patient prognosis (67, 68), indicating that the function of GLS2 may not be strictly suppressive.

**Figure 4:**
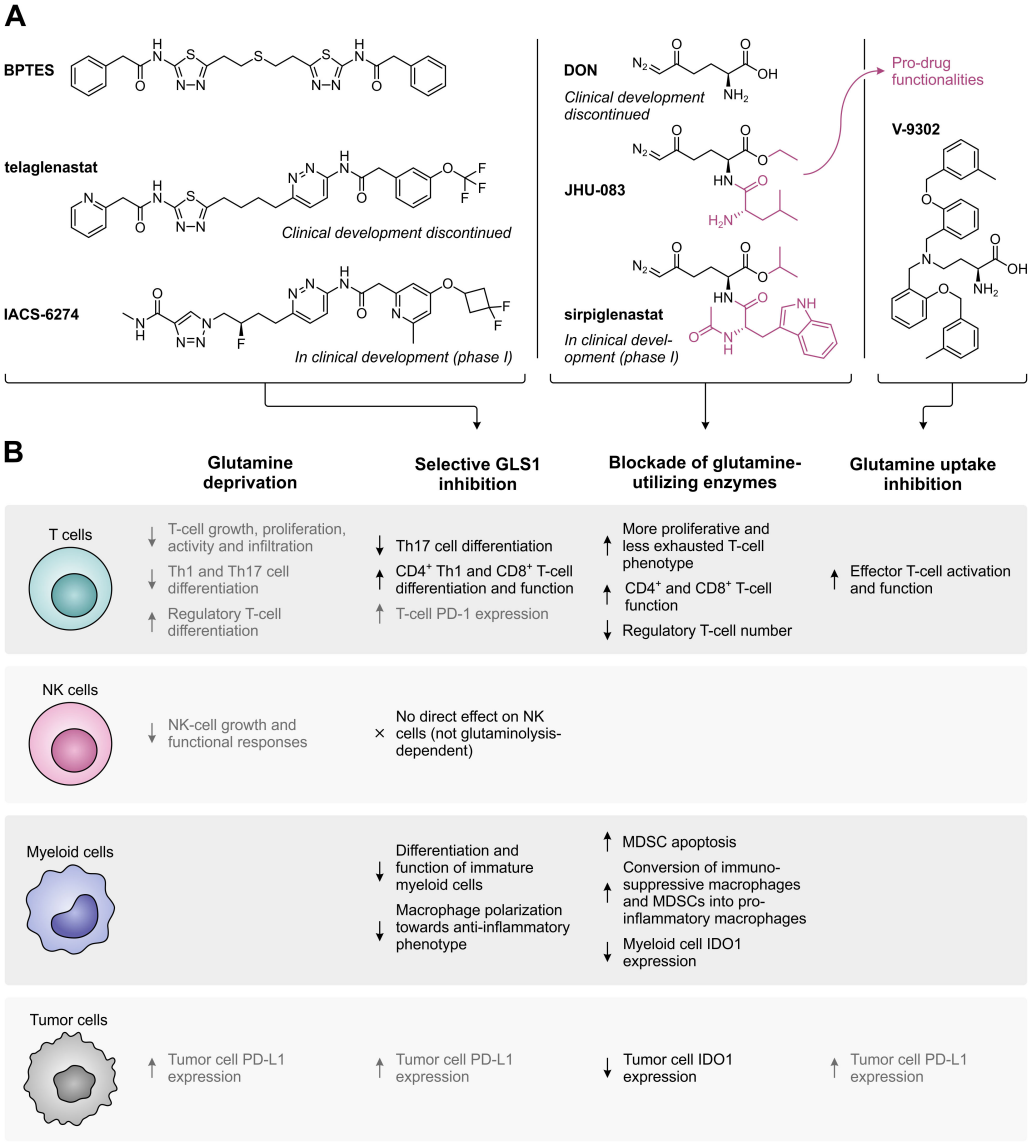
Overview of glutamine metabolism and uptake inhibitors, and their effects on different cell types in the tumor microenvironment. **(A)** Chemical structures of selective GLS1 inhibitors, broad-spectrum inhibitors of glutamine-utilizing enzymes, and a glutamine uptake inhibitor. For inhibitors which are currently or have previously been evaluated in clinical trials, the current status of clinical development is indicated. **(B)** Effects of glutamine deprivation and the different glutamine metabolism-targeting strategies on T cells, NK cells, myeloid cells and tumor cells. Effects shown in black have a positive impact on the anti-tumor immune response, whereas effects shown in grey have a negative effect.

Following the discovery that glutamine is an indispensable nutrient for the growth and survival of many cancer cells, several glutamine metabolism-targeting strategies have been evaluated as potential targeted anti-cancer therapies (**Figure 3B**). These include the selective, allosteric inhibition of GLS1 using bis-2-(5-phenylacetamido-1,2,4-thiadiazol-2-yl)ethyl sulfide (BPTES) (69) or its successors telaglenastat (CB-839) (70) and IACS-6274 (IPN60090) (**Figure 4A**, left) (71). Moreover, a number of competitive, irreversible inhibitors with broad-spectrum activity against glutamine-utilizing enzymes have been developed, also referred to as glutamine antagonists, which include 6-diazo-5-oxo-L-norleucine (DON) (72, 73) and its pro-drug successors JHU-083 (74) and sirpiglenastat (DRP-104) (**Figure 4A**, middle) (75). Finally, glutamine uptake can be competitively inhibited using the SLC1A5/ASCT2 transporter antagonist V-9302 (**Figure 4A**, right) (76). Starting clinical evaluation in the mid-1950s, DON has already demonstrated promising anti-tumor activity in various cancer types (77). However, clinical development of DON was discontinued due to significant toxicities. Telaglenastat has since entered clinical evaluation in 2014, but development was recently suspended after failure of two phase II clinical trials (ClinicalTrials.gov identifiers: NCT03428217 (78) and NCT04265534) and dissolution of developer Calithera Biosciences, Inc. More recently, IACS-6274 (NCT05039801) and sirpiglenastat (NCT06027086) entered clinical-stage development as well. Importantly, although the mentioned therapeutic strategies were initially solely perceived to exploit the dependence of tumors on glutamine, it has been increasingly recognized that their effects extend beyond those affecting the cancer cells, as will be discussed below.

In addition to directly supporting cancer cell proliferation, excessive tumoral metabolism of glutamine may also deprive tumor-infiltrating immune cells of an important nutrient. Similar to cancer cells, activated T cells upregulate glutamine transporters (including SLC1A5/ASCT2 and SLC38A1/SNAT1) and increase glutaminolysis to accommodate the demands of rapid proliferation (79–81). Moreover, sufficient levels of intracellular glutamine are required for activation of TCR-induced mTORC1 signaling (81, 82), as glutamine serves as a counter-substrate for import of the essential amino acid and mTORC1 activator leucine (**Figure 1A**) (83). Accordingly, glutamine-depriving conditions compromise the growth and proliferation of activated T cells *in vitro* (79, 80), whereas restoration of tumor interstitial glutamine levels by tumor-specific GLS1 knockout increases T-cell infiltration and activity *in vivo* (**Figure 4B**) (38). However, since different subsets of T cells engage distinct metabolic programs (21, 84), their dependency on glutamine availability also diverges. In particular, limitation of glutamine uptake inhibits the differentiation of naïve CD4^+^ T cells under Th1- and Th17-skewing conditions (82), while glutamine deprivation promotes their differentiation into CD4^+^CD25^+^FoxP3^+^ regulatory T cells (85, 86). These effects may at least partially be due to compromised *de novo* synthesis of α-ketoglutarate (85), nucleotides (86) or glutathione (87). In contrast to T cells, natural killer (NK) cells do not appear to require glutamine for fueling their metabolism, although glutamine deprivation does impair NK-cell growth and functional responses due to its role in the regulation of c-Myc expression (**Figure 4B**) (88).

Based on the evident role of GLS1 in cancer development, selective targeting of this enzyme presents an attractive therapeutic anti-cancer approach. This is supported by the direct anti-proliferative effect exerted by different GLS1 inhibitors on cancer cells of diverse origin in *in vitro* studies (70, 89, 90). However, inhibition of GLS1 may also indirectly affect tumor growth through the combined effect of elevated glutamine availability and inhibition of glutaminolysis on immune cell function. Notably, although both tumor cells and activated T cells upregulate glutaminolysis to fuel their proliferation (25, 79), the metabolism and proliferative ability of these cells is not equally disrupted by GLS1 inhibition (70, 91). More specifically, different subsets of T cells are differentially dependent on functional glutaminolysis, as genetic GLS1 disruption suppresses CD4^+^ T-cell differentiation into Th17 cells, but promotes CD4^+^ Th1 and cytotoxic CD8^+^ T-cell differentiation and function (**Figure 4B**). This is associated with the altered epigenetic regulation of gene expression caused by modulation of α-ketoglutarate levels (84). Furthermore, restoration of glutamine availability upon GLS1 inhibition restores NK-cell cytotoxicity, which is not abolished by abrogation of NK-cell glutaminolysis (88). In contrast, selective inhibition of GLS1 suppresses the differentiation and immunosuppressive function of immature myeloid cells as well as the polarization of macrophages towards an anti-inflammatory phenotype (**Figure 4B**) (92–94).

Despite the complementary anti-proliferative effect exerted by GLS1 inhibitors on tumor cells and their stimulatory effect on the immune cell compartment, monotherapy with BPTES or telaglenastat has demonstrated variable efficacy in *in vivo* models (70, 90, 91, 93, 95). Moreover, only limited single-agent activity has been observed for telaglenastat in clinical trials (96, 97), which may be due to upregulation of compensatory metabolic pathways in the targeted cancer cells (95, 98, 99). Broader blockade of glutamine-utilizing enzymes or inhibition of glutamine uptake may at least partially resolve these issues, although they would logically also disrupt the metabolism of immune cells. Nonetheless, inhibition of glutamine-utilizing enzymes by JHU-083 or sirpiglenastat does not disable the *in vivo* anti-tumor immune response, but instead conditions T cells towards a more proliferative, less exhausted phenotype (**Figure 4B**) (74, 75, 100). This is attributed to the remarkable flexibility of T cells, but not tumor cells, to use glucose for replenishment of TCA cycle intermediates when glutamine metabolism is blocked (74). Furthermore, sirpiglenastat enhances CD4^+^ and CD8^+^ T-cell function and decreases regulatory T-cell numbers (100), while JHU-083 promotes anti-tumor immunity by inducing apoptosis of MDSCs, stimulating the conversion of immunosuppressive macrophages and MDSCs into pro-inflammatory macrophages, and inhibiting the expression of IDO1 in both myeloid and tumor cells (101). Inhibition of glutamine uptake by SLC1A5/ASCT2 inhibitor V-9302 additionally induces a marked reduction of tumor growth *in vivo*, which is accompanied by enhanced activation and functionality of effector T cells (**Figure 4B**). Compensatory upregulation of the glutamine transporter SLC6A14/ATB^0,+^ by T cells, but not tumor cells, is suggested to explain this unanticipated effective immune response (38).

The effectiveness of GLS1 inhibition may also be limited by the exhaustion of T cells *in vivo*, as GLS1 deficiency over time induces expression of PD-1 on T cells (84). Moreover, inhibition of GLS1, as well as glutamine deprivation or inhibition of glutamine uptake, results in upregulation of tumoral PD-L1 expression (**Figure 4B**) (102, 103). These observations indicate that dual targeting of glutamine metabolism and the PD-1/PD-L1 interaction may improve the therapeutic anti-tumor response. Accordingly, α-PD-1 or α-PD-L1 treatment, as well as blockade of CTLA-4, enhances the efficacy of inhibitors targeting GLS1 (91, 102), glutamine-utilizing enzymes (74, 75) or glutamine uptake *in vivo* (104). Moreover, inhibition of GLS1 or glutamine-utilizing enzymes enhances the response to immune checkpoint inhibitors in immune checkpoint blockade-resistant mouse models (93, 101). In a phase II clinical trial, telaglenastat combined with the PD-1 inhibitor nivolumab showed a modest objective response rate in α-PD-1/PD-L1-refractory melanoma patients based on preliminary results (NCT02771626) (105). In contrast, a phase II study of telaglenastat in combination with pembrolizumab (α-PD-1) and chemotherapy in patients with metastatic non-small cell lung cancer was terminated due to lack of clinical benefit (NCT04265534). Clinical evaluation of sirpiglenastat combined with durvalumab (α-PD-L1), for which a phase I/II trial in advanced stage fibrolamellar hepatocellular carcinoma patients has recently been initiated (NCT06027086), may provide further clarity on the effectiveness of combining glutamine metabolism-targeting strategies with immune checkpoint blockade.

In conclusion, GLS1 inhibition and other glutamine metabolism-targeting strategies have presented themselves as promising approaches for cancer treatment, both through direct targeting of glutamine-addicted tumor cells and through enhancement of the anti-tumor immune response. The latter effect appears to be at least partially owed to the plasticity of T cells to accommodate perturbations in their glutamine metabolism, although further efforts are required to completely understand the mechanisms underlying this favorable phenomenon. Moreover, as the discussed approaches have yet to demonstrate convincing efficacy in clinical trials, a rational exploration of the drug combination space may prove valuable for future clinical endeavors.

## 4 The arginine-metabolizing enzyme ARG1

Similar to glutamine, arginine is classified as a conditionally essential amino acid, as it must be provided through nutrition during conditions of stress as well as during fetal and neonatal development (106). It is a highly versatile amino acid, serving as a precursor for the synthesis of proteins, other amino acids and a variety of biologically important metabolites, including nitric oxide (NO), creatine, agmatine and polyamines (**Figure 1B**). The metabolic fate of arginine is determined by the coordinated action of a diverse set of highly regulated enzymes and arginine transporters (107). Among these are the arginase (ARG) enzymes, catalyzing the hydrolysis of arginine into the non-proteinogenic amino acid ornithine and the waste product urea. This metabolic conversion presents the final step of the urea cycle for ammonia detoxification and provides ornithine as a substrate for polyamine synthesis and interconversion into proline or glutamate (107).

In humans, ARG is expressed as two isoforms differing in subcellular localization and distribution among cell and tissue types (108). ARG1 is a cytosolic enzyme predominantly and abundantly expressed in the liver as a key enzyme of the urea cycle, although it is also expressed by cells of the myeloid lineage to regulate immune responses (109–111). In contrast, mitochondrial ARG2 has a more ubiquitous, extrahepatic expression pattern (109), and is suggested to primarily function as a regulator of arginine availability (112). Similar to ARG1, it can also be expressed by various immune cells (113–116), although its role in the immune system is still largely elusive. In accordance with their physiological tissue distribution, expression of ARG1 by tumor cells is mainly limited to hepatocellular carcinoma (117, 118), while ARG2, although largely understudied compared to ARG1, is found in the neoplastic cells of several human cancer types (39, 119–121). Importantly, however, and in contrast to the GLS1 enzyme, the main immunosuppressive activity exerted by ARG enzymes does not stem from their expression by tumor cells, but rather from ARG1 expressed by tumor-infiltrating immune cells.

**Figure 5:**
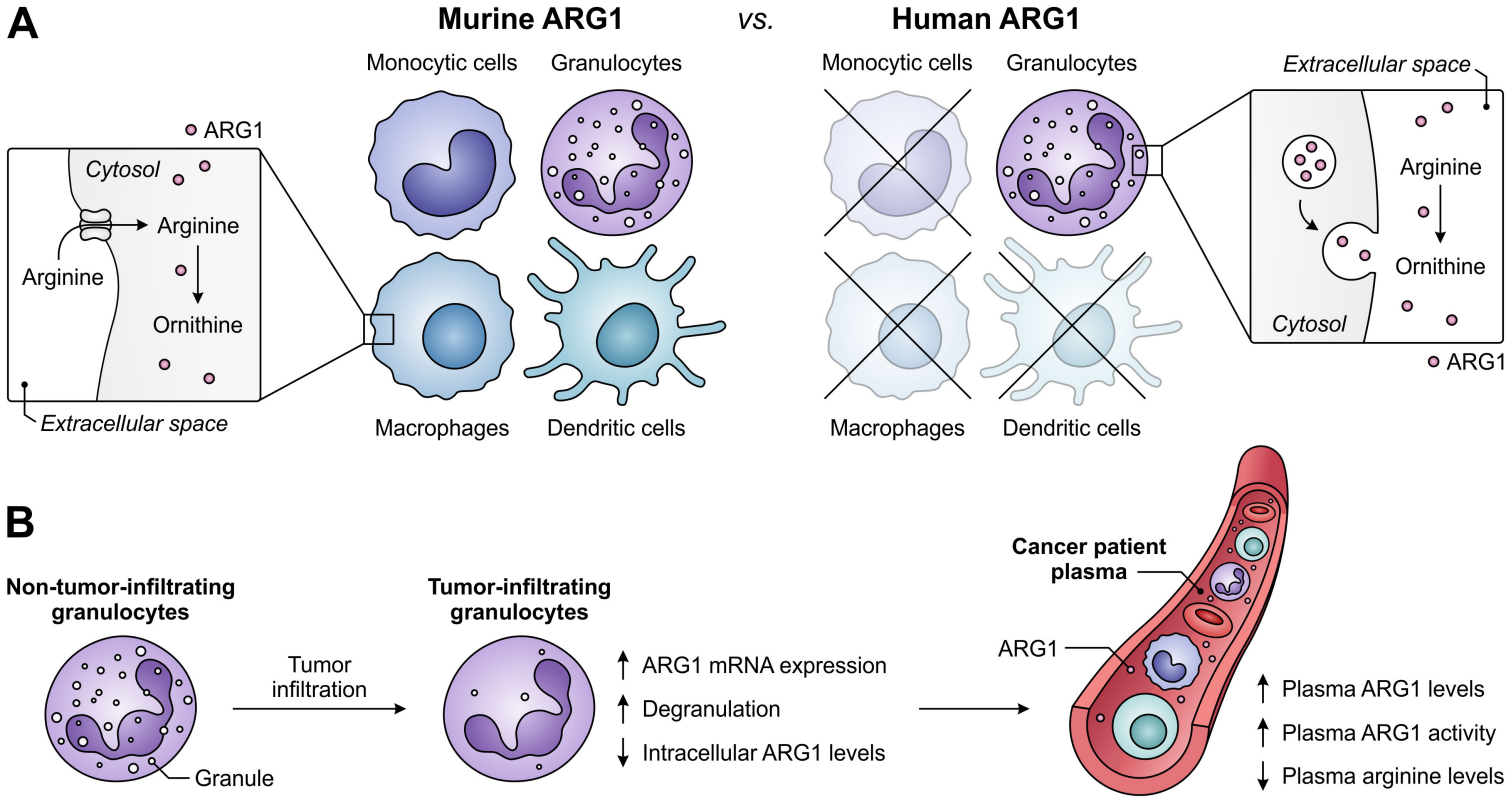
Expression of ARG1 by the murine and human myeloid compartment, and ARG1-related effects of granulocyte tumor-infiltration in cancer patients. **(A)** Difference between ARG1 expression and site of activity in murine *versus* human myeloid cells. Murine ARG1 is expressed in various myeloid cell types and acts predominantly intracellularly, whereas human ARG1 is solely expressed by granulocytes, which store ARG1 in granules to be released for extracellular arginine degradation. **(B)** Effects of human granulocyte infiltration into tumors on the expression, granular release and activity of ARG1 in cancer patients.

A role for ARG1 in the immune system was first identified based on its Th2-type cytokine-inducible expression in various murine myeloid cell types, including macrophages and dendritic cells (122, 123). However, the cell-type specificity and inducibility of ARG1 expression in myeloid cells differs considerably between humans and mice (**Figure 5A**) (124), complicating the translation from murine studies to human subjects. In contrast to its murine counterpart, human ARG1 is strictly and mostly constitutively expressed by granulocytes, including neutrophils, granulocytic MDSCs (G-MDSCs) and eosinophils (125, 126). For this reason, murine ARG1-expressing myeloid cells other than granulocytes will not receive focus in this review. However, this is not where the discrepancies end, as murine myeloid cells mostly regulate extracellular arginine levels through its uptake and subsequent ARG1-mediated degradation (28). In contrast, human granulocytes store ARG1 in their granules to become active only upon exocytosis (**Figure 5A**) (110, 111, 125, 127), which is reported to involve a proteolytic cleavage step (110, 111). Finally, murine G-MDSCs have been found to release ARG1 in small extracellular vesicles (128), which is a phenomenon currently only observed for ARG1-expressing tumor cells in the human setting (129).

In patients with various cancer types, high ARG1 expression and activity is found in both circulating and tumor-infiltrating myeloid cells, with G-MDSCs as its major source (29, 117, 130–132). Notably, granulocytes of glioblastoma patients are found to be in a degranulated state, while plasma ARG1 levels of these patients are significantly increased (**Figure 5B**) (133). Furthermore, tumor-infiltrating granulocytes in patients with non-small cell lung cancer or renal cell carcinoma have decreased ARG1 levels compared to their non-tumor-infiltrating counterparts (126, 134), despite having increased ARG1 mRNA expression (126). These findings indicate the tumor-associated release of granule-stored ARG1 in cancer patients, which is in line with the elevated ARG1 levels and activity, as well as decreased arginine levels, found in the plasma of patients with diverse tumor types (**Figure 5B**) (29, 111, 117, 126, 134). Importantly, since the release of ARG1 occurs upon tumor infiltration (134), arginine levels in the TME may be even further reduced. In murine pancreatic tumors, near-complete depletion of arginine is detected in tumor interstitial fluids, whereas ornithine levels are increased compared to those in plasma samples (135). Although ARG1 expression and its mode of action differs between the murine and human myeloid compartment, this suggests that myeloid cell-expressed ARG1 can efficiently deprive the TME of a valuable nutrient.

**Figure 6:**
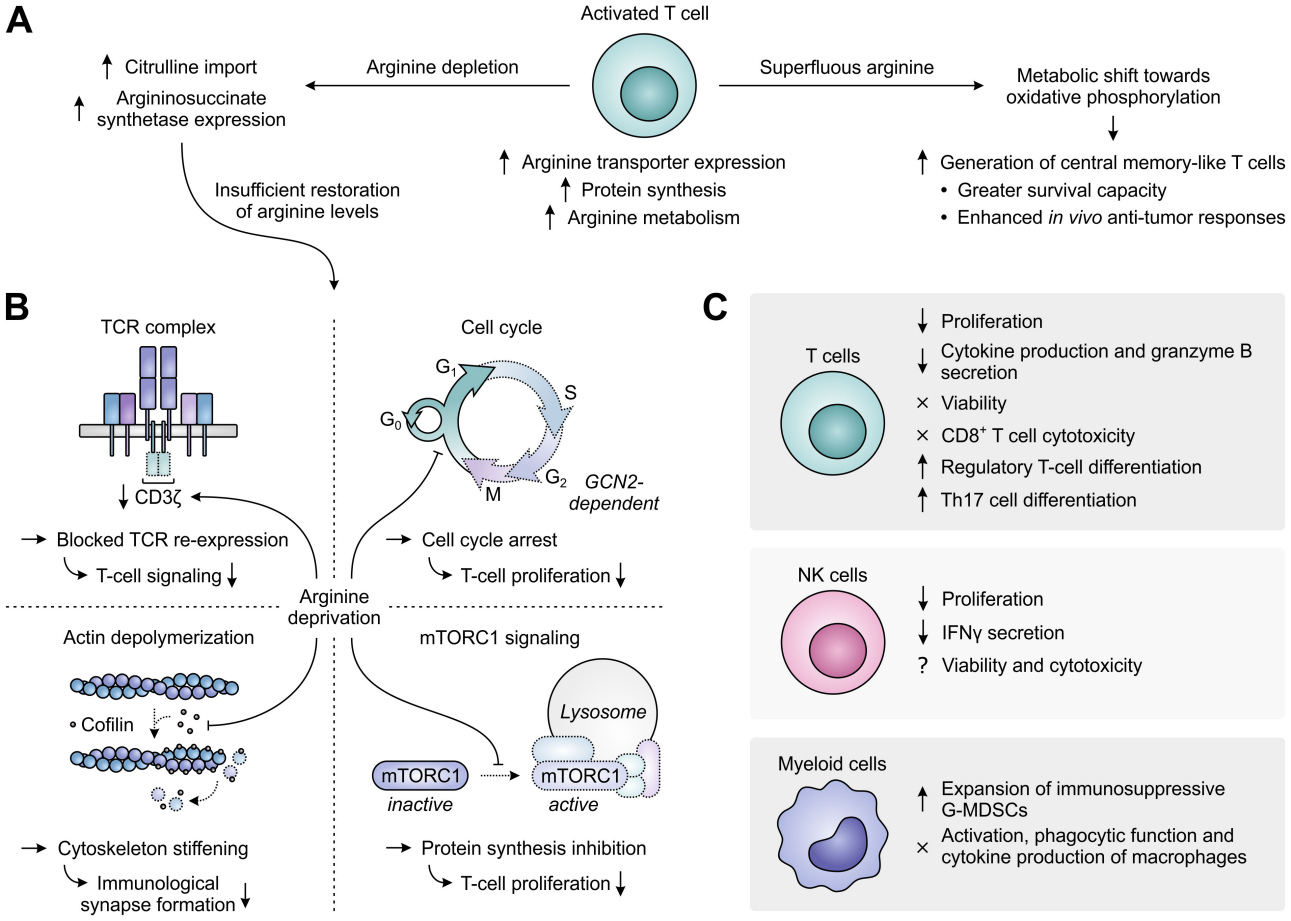
Regulation of immune cell function by arginine availability. **(A)** Arginine-associated changes in T cells upon their activation (center), and effects of arginine abundance (right) *versus* depletion (left) on T-cell metabolism and function. **(B)** In the case of persisting arginine deprivation, T-cell proliferation and function are affected through various mechanisms. These include the downregulation of CD3ζ expression resulting in blocked T-cell receptor (TCR) re-expression (upper left), the general control nonderepressible 2 (GCN2)-dependent arrest of cell cycle progression (upper right), the global reduction in protein synthesis upon inhibition of mammalian target of rapamycin complex 1 (mTORC1) signaling (lower right) and the stiffening of the cytoskeleton due to inhibition of the actin-depolymerizing factor cofilin (lower left). Dotted outlines and arrows indicate components and processes that are downregulated or inhibited. **(C)** Effects of arginine deprivation on different immune cell types in the tumor microenvironment.

Enhanced activity of ARG1 in the TME may directly support tumor growth by supplying tumor cells with ornithine or ornithine-derived polyamines, which are essential for cell growth and proliferation (136, 137), and by decreasing cytotoxic NO production (137). However, these are not the sole mechanisms through which ARG1 promotes tumor growth, as its activity can also adversely affect the activation and function of tumor-infiltrating immune cells. In activated T cells, arginine is not only required to keep up with the fast rate of activation-induced protein synthesis, but it is also rapidly metabolized by virtue of the upregulation of ARG2 and that of other enzymes determining its downstream fate, including conversion into the polyamine precursors agmatine and putrescine (138). To meet the high demand for arginine, activated T cells mostly depend on its enhanced uptake, which is achieved through upregulation of the arginine transporter SLC7A1/CAT-1 (**Figure 6A**) (138, 139). When extracellular arginine is superfluous, activated T cells shift their metabolism from aerobic glycolysis towards oxidative phosphorylation through sensing of arginine levels by different transcriptional regulators. While this limits their differentiation, it instead favors the generation of central memory-like T cells with greater survival capacity and enhanced *in vivo* anti-tumor responses (138). In contrast, when extracellular levels of arginine are depleted, intracellular arginine levels become insufficient, despite attempts of T cells to restore them by increasing the import of its precursor citrulline and upregulating the expression of the arginine biosynthetic enzyme argininosuccinate synthetase (**Figure 6A**) (140, 141).

Arginine-depriving conditions or ARG1-expressing cells profoundly inhibit the activation-induced proliferation of T cells (126, 142, 143). Over the years, a number of mechanisms underlying this inhibitory effect have already been elucidated (**Figure 6B**). A prime mechanism is the reversible reduction of CD3ζ chain expression, which blocks cell surface re-expression of the TCR complex after its antigen-induced internalization and thus compromises T-cell signaling (142, 144). Accordingly, decreased CD3ζ expression is found in the peripheral or tumor-infiltrating T cells of cancer patients with high myeloid ARG1 expression (28, 29). Arginine starvation also disrupts the cell cycle progression of activated T cells, arresting them in the G_0_/G_1_ phase and thereby hampering their proliferation (145). Cell cycle arrest is caused by arginine deprivation-induced activation of the GCN2 pathway, which blocks the upregulation of critical cell cycle progression regulators by promoting a global arrest in *de novo* protein synthesis (145, 146). Amino acid sensing by mTORC1 may additionally be involved in the effects of arginine starvation on T-cell proliferation (147, 148). Finally, the actin-depolymerizing factor cofilin is inhibited in T cells cultured under arginine-depriving conditions. This impairs formation of the immunological synapse between T cells and antigen-presenting cells due to stiffening of the actin cytoskeleton and thereby hampers effective T-cell activation (149).

Besides affecting T-cell activation and proliferation, arginine depletion also specifically inhibits the T-cell production of several cytokines, including IFNγ, IL-5 and IL-10, which is associated with their reduced mRNA expression, and secretion of the cytotoxic protease granzyme B (**Figure 6C**) (142, 150). In contrast, T-cell chemotaxis and the antigen-specific cytotoxicity of CD8^+^ T cells are largely preserved (150, 151). Moreover, the viability of T cells remains unaltered upon arginine depletion (142–144), allowing these cells to re-gain their proliferative and cytokine secretory potential upon arginine replenishment and re-stimulation (143). Arginine deprivation also induces the generation of CD4^+^CD25^+^FoxP3^+^ regulatory T cells, either from naïve CD4^+^ T cells or from a pre-existing natural CD4^+^CD25^+^FoxP3^−^ regulatory T cell population (147, 152–154). This may require or be potentiated by TGF-β (147, 154) and involves the mTORC1 signaling pathway (147). However, ARG1-expressing MDSCs may also promote Th17 differentiation of naïve CD4^+^ T cells (155), indicating a complex role for arginine in T-cell differentiation. Depletion of arginine additionally impairs NK-cell proliferation and IFNγ secretion, while effects on NK-cell viability and cytotoxicity are still unclear based on the existence of contradictory reports (117, 156–158). Moreover, arginine deprivation promotes the expansion of immunosuppressive G-MDSCs in mice (159), while it does not affect the activation, phagocytic functions or cytokine production of murine macrophages (**Figure 6C**) (160).

**Figure 7:**
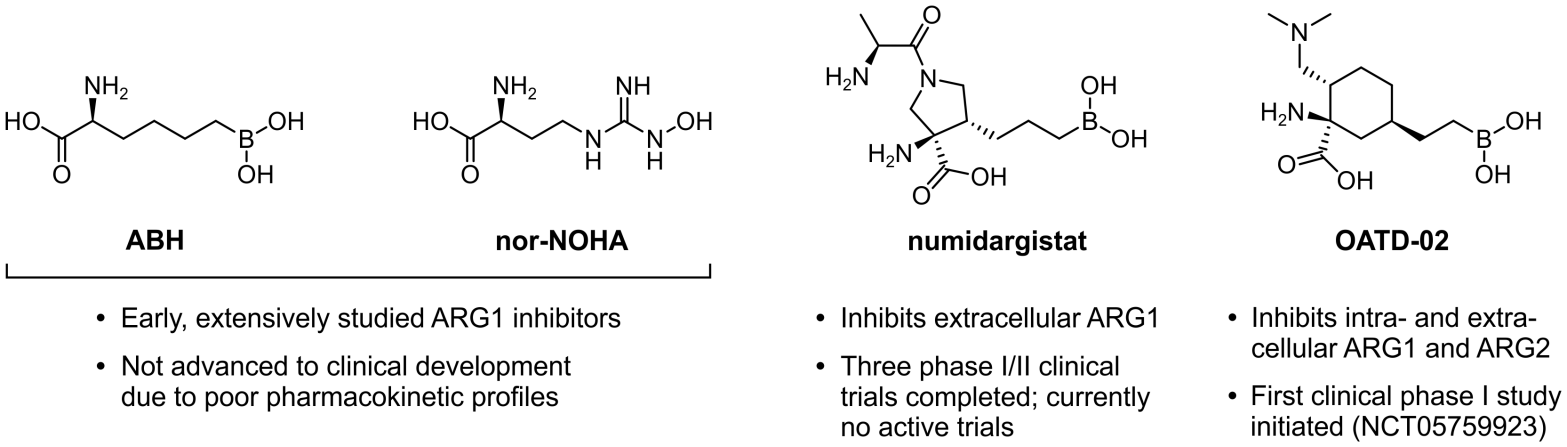
Chemical structures of ARG1 inhibitors and their current status of clinical development.

In an effort to constrain the immunosuppressive effects exerted by ARG1-expressing myeloid cells, several ARG-targeting small molecule inhibitors have been developed. These are limited to only dual inhibitors of ARG1 and ARG2, as the highly conserved active sites of these enzymes encumber the development of isoform-specific variants (161). The extensively studied (2*S*)-2-amino-6-boronohexanoic acid (ABH) and *N* ^ω^-hydroxy-nor-l-arginine (nor-NOHA) were among the first inhibitors to be identified (**Figure 7**) (162, 163), but poor pharmacokinetic properties have limited their clinical application (164, 165). More recently, inhibitors with considerably improved pharmacokinetic profiles have been developed, which include numidargistat (INCB001158; CB-1158) (117, 166) and OATD-02 (OAT-1746) (**Figure 7**) (167). Whereas OATD-02 can inhibit both intracellular and secreted ARG enzymes (167), numidargistat acts only extracellularly due to its inefficient ability to cross the cell membrane (117). Notably, this spares not only the activity of crucial liver-expressed ARG1, but also that of ARG2, which appears to function only within the cell. However, consequences of ARG2 inhibition may be limited based on the phenotype of ARG2-deficient mice (168). Furthermore, *in vitro* studies indicate that inhibition of ARG2 may even be beneficial for the anti-tumor T cell response (115, 169), whereas it may also directly inhibit the growth of ARG2-expressing tumors (170). Alternative approaches to directly or indirectly inhibit ARG activity have also been reported, which include isoform-specific ARG antibodies (171, 172) and therapeutic peptide vaccines (173, 174).

ARG inhibition or myeloid cell ARG1 knock-out inhibits tumor growth in various syngeneic mouse models, which has compellingly been associated with changes of the TME immune cell composition towards a tumor-hostile environment (28, 117, 151, 170, 175, 176). Moreover, in different murine tumor models, inhibition of ARG enhances the therapeutic efficacy of α-PD-(L)1 treatment (117, 151, 177). Based on these promising results, numidargistat has entered a number of phase I/II clinical trials over the last several years. These include two completed trials in patients with advanced solid tumors in which numidargistat was studied as monotherapy and in combination with the PD-1 inhibitor pembrolizumab (NCT02903914) or retifanlimab (NCT03910530). Preliminary phase I data reported for the former demonstrate a slight improvement in the objective response rate of colorectal carcinoma patients treated with numidargistat mono- or combination therapy compared to historical control data, and an increased number of intratumoral CD8^+^ T cells in post-treatment biopsies (178). However, further clinical results will have to be awaited to draw any firm conclusions on the benefit of ARG inhibition for cancer treatment. The clinical evaluation of OATD-02, which has just recently entered its first phase I trial in patients with advanced and/or metastatic solid tumors (NCT05759923), should also contribute to this quest.

Taken together, the growing body of research on the role of myeloid cell-expressed ARG1 in cancer immune escape underscores the promise of this enzyme as a target for cancer immunotherapy. To date, this is mostly supported by *in vitro* studies performed on human immune cells and *in vivo* studies with syngeneic mouse models. Importantly, however, it remains to be determined whether the differential expression of ARG1 between mice and humans poses any problem for the translation of this approach towards cancer patients. For this, data from clinical trials is pivotal, and these data should also provide insight as to whether extracellularly-restricted ARG inhibitors may be more beneficial for cancer treatment compared to inhibitors acting also intracellularly, or *vice versa*.

## 5 The arginine-metabolizing enzyme iNOS

A different metabolic fate of arginine is its conversion into the important signaling molecule NO. This reaction is catalyzed by nitric oxide synthase (NOS) enzymes and yields citrulline as a by-product that can be recycled back into its precursor arginine (**Figure 1C**). Three distinct isoforms of NOS are encoded by the mammalian genome, which are neuronal NOS (nNOS or NOS1), inducible NOS (iNOS or NOS2) and endothelial NOS (eNOS or NOS3). Both nNOS and eNOS are constitutively expressed enzymes that can be triggered by calcium influx to transiently produce nanomolar concentrations of NO (179). A calcium-independent increase in eNOS activity can additionally be elicited by eNOS phosphorylation (180). Under physiological conditions, nNOS plays a fundamental role in neurotransmission, while eNOS is a critical regulator of various cardiovascular functions, including vasodilation (179). Distinctively, expression of iNOS can be induced in a variety of cell types upon exposure to a broad range of factors, including pro-inflammatory cytokines and hypoxia (181, 182), while it is concurrently subject to intricate regulation by NO levels and arginine availability (183–185). iNOS is capable of calcium-independently producing sustained micromolar levels of NO, through which it primarily supports pathogen killing and regulation of immune responses (179).

**Figure 8:**
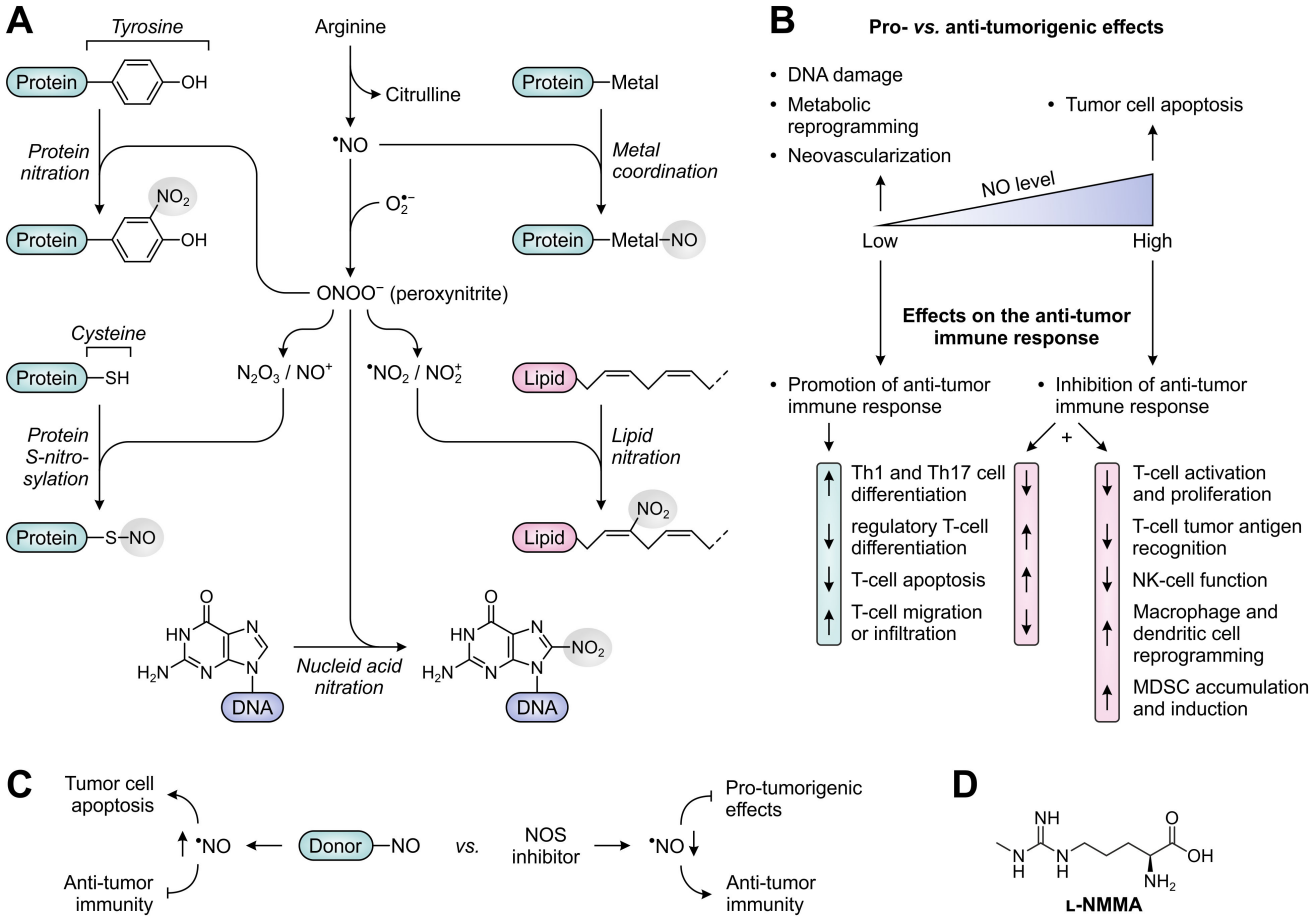
Molecular and cellular effects of nitric oxide (NO), and therapeutic strategies to alter NO levels for cancer treatment. **(A)** Reactivity of NO and NO-derived species with biomolecules. NO can directly alter protein function through metal coordination (top right), whereas various NO-derived species can nitrate and *S*-nitrosylate proteins (left) as well as nitrate lipids and DNA (lower right and bottom). **(B)** Effects of low and high concentrations of NO on tumor development (top) and the anti-tumor immune response (bottom). **(C)** NO-donors and NOS inhibitors as opposing strategies to alter NO levels and thereby affect tumor development and the anti-tumor immune response. **(D)** Chemical structure of the pan-NOS inhibitor l-*N* ^G^-monomethyl-arginine (l-NMMA).

NO, the primary product of NOS activity, is a short-lived, highly diffusible free radical (frequently denoted as ^•^NO) capable of freely crossing cellular membranes (**Figure 8A**). While NO is relatively unreactive towards most biomolecules, it can very rapidly form reactive nitrogen species (RNS) by reacting with molecules having unpaired electrons, such as other free radicals and transition metal ions (186). An important reaction partner of NO is superoxide anion ( ${\text{O}}_{2}^{•-}$ ), which is formed by NOS enzymes upon depletion of the substrate arginine or co-factor tetrahydrobiopterin (BH_4_) (187), but can also be generated by other sources such as NADPH oxidase (188). Reaction of NO with superoxide anion yields the powerful, but short-lived oxidant peroxynitrite (ONOO^−^) (**Figure 8A**), which can efficiently nitrate protein tyrosine residues upon its decomposition (186). Other NO-derived RNS (*i.e.*, N_2_O_3_ and NO^+^) can readily react with the thiol side chain of cysteine residues to yield protein *S*-nitrosylation (189). Both nitration and *S*-nitrosylation are highly selective processes, and have the potential to greatly alter the structure and function of target proteins (186, 189). In addition to protein modification, NO-derived RNS can cause DNA damage by modifying nucleic acids (190), and can generate nitrolipids capable of activating cell-signaling pathways (191). Moreover, NO can coordinate to transition metals bound by enzymes and transcription factors, thereby altering their activity (**Figure 8A**) (192). As a relevant exemplar, coordination of NO to the heme-bound iron of soluble guanylate cyclase (sCG) induces production of the second messenger cyclic guanosine monophosphate (cGMP), which serves to regulate various physiological processes (193, 194).

It is broadly acknowledged that NO plays an important role in both cancer development and subsequent progression. NO-producing NOS enzymes are aberrantly expressed in a variety of human tumors (195), with effects of elevated NO levels being dependent on the cellular source, concentration, local chemical environment and cellular target (196). Among the three isoforms, iNOS has most extensively been studied in cancer based on its frequent detection in tumor cells, tumor-infiltrating immune cells and tumor-associated fibroblasts. Overexpression of eNOS has additionally been found in the vascular endothelial cells of a myriad of cancer types (195). Although the elevated expression of NOS enzymes, particularly iNOS, has recurrently been associated with tumor malignancy and poor patient prognosis, it is increasingly recognized that the role of NO in cancer is inherently complex owing to its diverse spectrum of cellular sources and biological effects (196). Notably, NO acts as a double-edged sword in cancer by predominantly exerting pro-tumorigenic effects at relatively low concentrations, such as inducing DNA damage, tumor cell metabolic reprogramming and neovascularization, while suppressing tumor growth at higher concentrations by inducing apoptosis (**Figure 8B**) (195, 197). Furthermore, dependent on its concentration, NO can either stimulate or suppress the anti-tumor immune response, as will be discussed below.

Early research has indicated that NO promotes an effective immune response when present at low, physiological concentrations. This is for instance achieved through the cGMP-dependent induction of Th1- and Th17-cell differentiation (198, 199), the suppression of regulatory T-cell generation (200), and the inhibition of T-cell apoptosis through caspase *S*-nitrosylation (**Figure 8B**) (201). However, within the TME, the production of NO is often considerably enhanced due to the presence of iNOS-expressing tumor cells, macrophages and MDSCs, resulting in NO concentrations capable of significantly hampering immune responses. At elevated concentrations, NO inhibits T-cell activation and proliferation by impeding activation-induced protein tyrosine phosphorylation, which may occur through nitration or *S*-nitrosylation of crucial protein residues, or through a cGMP-dependent pathway (**Figure 8B**) (202–207). In addition, these and other NO-dependent mechanisms can prime T cells to undergo apoptosis (204, 208, 209). High concentrations of NO also suppress the *in vitro* polarization of CD4^+^ T cells towards both Th1 and Th17 phenotypes (210–213), while an effective immune response may further be precluded by the NO-induced generation of CD4^+^CD25^+^FoxP3^−^ regulatory T cells (214). Moreover, besides affecting the functionality of T cells, high concentrations of NO can impair effector functions of NK cells through nitration of crucial signaling proteins (215), and induce phenotypic and metabolic reprogramming of macrophages and dendritic cells (**Figure 8B**) (216–218).

**Figure 9:**
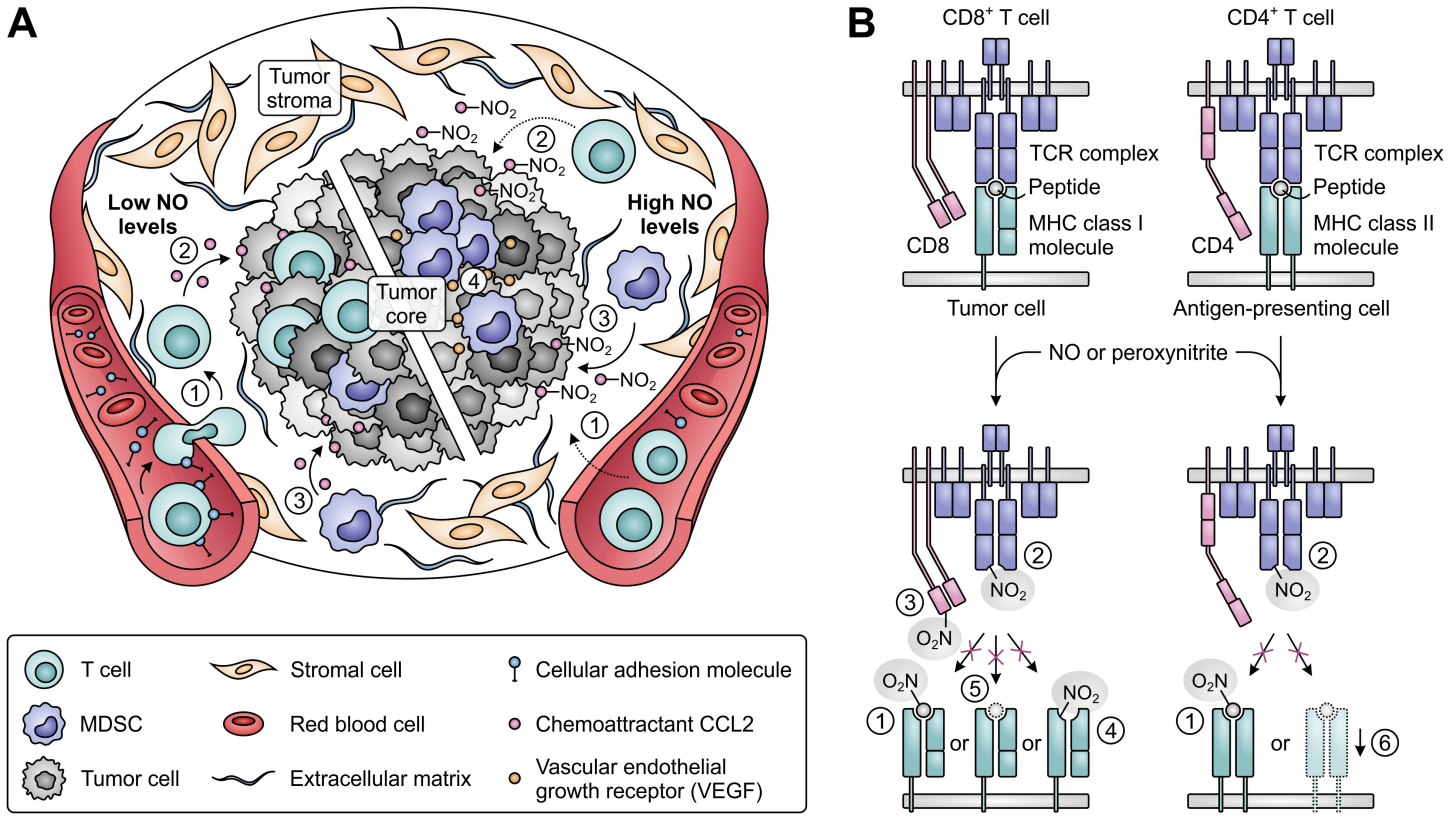
Effects of nitric oxide (NO) and peroxynitrite levels on tumor immune cell infiltration and T-cell antigen recognition. **(A)** Effect of low (left) and high levels of NO (right) on the migration and infiltration of T cells and MDSCs into the tumor. High concentrations of NO suppress migration of T cells into tumors [1], and nitration of the chemoattractant CCL2 further precludes T-cell infiltration into the tumor core [2]. MDSC recruitment into the tumor core is not affected by CCL2 nitration [3], whereas MDSC accumulation and induction is promoted by iNOS-induced upregulated VEGF [4]. **(B)** Effects of NO-derived peroxynitrite on the recognition of antigens on tumor and antigen-presenting cells by T cells. Peroxynitrite can hinder recognition through nitration of MHC class I- or II-presented peptides [1], nitration of TCR [2] and CD8 molecules on T cells [3], nitration of MHC class I molecules [4], downregulation of antigenic peptide generation through inhibition of proteasomal activity [5] and downregulation of MHC class II gene transcription [6].

An effective immune response also relies on the ability of T cells to infiltrate tumor tissues and to successfully recognize their cognate tumor antigens (**Figure 9**). While low concentrations of NO stimulate the expression of cellular adhesion molecules mediating the migration of T cells from blood vessels into the tumor stroma (219), high concentrations act suppressive, thereby hampering the infiltration of T cells into tumor tissues (**Figure 9A**) (219–221). Moreover, NO-induced nitration of the chemoattractant CCL2 can cause successfully migrated T cells to remain trapped in the tumor stroma (222). In contrast, nitrated CCL2 has an unaltered ability to recruit MDSCs into the tumor core (222), while accumulation and induction of MDSCs is further promoted by iNOS-dependent upregulation of vascular endothelial growth factor (VEGF) secretion (**Figure 9A**) (223). NO-mediated nitration can also abrogate the recognition of tumor antigens by T cells (**Figure 9B**), as nitration of even a single tyrosine residue in major histocompatibility complex (MHC)-presented peptides can hinder their interaction with the TCR (224, 225). Moreover, MDSCs from peripheral lymphoid organs can induce nitration of TCR and CD8 molecules on T cells upon antigen-specific cell–cell contact, rendering them insensitive to stimulation by the presented antigen (226, 227). Tumor-infiltrating MDSCs, which greatly upregulate iNOS expression in response to the hypoxic TME, can additionally affect nearby cells without requiring antigen-specific interaction (228). This allows them to nitrate MHC class I molecules on neighboring tumor cells, thereby disturbing their peptide presentation and allowing them to become resistant to antigen-specific cytotoxic T cells (229). Furthermore, NO-derived peroxynitrite can inhibit proteasomal activity in tumor cells, resulting in decreased generation of antigenic peptides (230). Finally, through inducing the downregulation of MHC class II gene transcription, NO can also negatively affect the function of antigen-presenting cells (**Figure 9B**) (231).

Based on the pivotal role of NO in cancer development, different therapeutic strategies aimed at the modulation of NO levels have already been considered for cancer treatment. Noteworthy in this context is the clear dichotomy presented by the assessment of both NO-donating molecules and NOS inhibitors. NO donors are studied for their ability to directly induce the apoptosis of tumor cells or to sensitize them to other therapies (232), but they will evidently frustrate the immune system as well. In contrast, NOS inhibitors may serve as targeted therapies to revert the pro-tumorigenic effects of NO on tumor cells (233), while they may also reinvigorate anti-tumor immune responses (**Figure 8C**). During the recent decades, a large number of NOS inhibitors has already been developed and evaluated in clinical trials for various disease indications. These include both pan- and isozyme-selective inhibitors of the NOS enzymes (234). In murine cancer models, treatment with either type of inhibitor has been shown to reduce tumor growth (30, 233, 235, 236), but availability of *in vivo* data on the contribution of the immune system to this effect is still very limited (235, 237). Since peroxynitrite may be the major NO-derived effector molecule responsible for T-cell dysfunction, peroxynitrite neutralization or blockade of its formation may also present an attractive therapeutic strategy, which has already demonstrated efficacy in murine models (207, 222). Only recently, the first phase I/II clinical trial evaluating the use of an NOS inhibitor for anti-cancer therapy has been completed (NCT02834403). Results from this trial demonstrate a promising efficacy for the pan-NOS inhibitor l-*N* ^G^-monomethyl-arginine (l-NMMA; **Figure 8D**) in combination with docetaxel chemotherapy in triple-negative breast cancer patients, and show modest differences in circulating immune cell composition between responders and non-responders (238). Moreover, l-NMMA is currently being evaluated in a phase I trial in combination with pembrolizumab (α-PD-1) in patients with different solid tumors (NCT03236935), which may yield further clarification on the effects of NOS inhibition on the anti-tumor immune response.

Overall, there is accumulating evidence that the complex, multifaceted role of iNOS in cancer includes the facilitation of tumor immune escape. Distinctive from the action of other amino acid-metabolizing enzymes, this may not only involve the direct suppression of T-cell responses, but also the impediment of their tumor infiltration and antigen recognition. However, as *in vivo* data on the effects of NOS inhibitors on the anti-tumor immune response are currently still largely lacking, attention should be directed towards studying these inhibitors in more complex models. Simultaneously, such studies can contribute to our understanding of whether the use of either pan- or isozyme-selective NOS inhibitors should be the preferred approach for future clinical trials.

## 6 The tryptophan-metabolizing enzymes IDO1 and TDO

Unlike glutamine and arginine, tryptophan is an essential amino acid that is exclusively obtained by humans through dietary intake. In addition to serving as a fundamental protein building block, tryptophan is a precursor for various bioactive compounds. These include metabolites generated along the serotonin pathway and indoles produced by the gut microbiota or by host cells (**Figures 1D, E**) (37, 239). However, the vast majority of tryptophan degradation occurs through the kynurenine pathway, which is initiated and rate-limited by the paralogous enzymes IDO1 and IDO2, and the evolutionarily unrelated TDO. Each of these enzymes catalyzes the oxidation of tryptophan to yield *N*-formylkynurenine, which is then rapidly hydrolyzed to kynurenine and can be further metabolized into an array of downstream molecules (**Figure 1D**) (240).

Expression of IDO1, the most extensively studied enzyme of the kynurenine pathway, is highly inducible across a broad range of cell and tissue types, with IFNγ serving as its main inducer (241, 242). While initially recognized for its role in the defense against infectious pathogens (243), IDO1 is now widely acknowledged as a critical regulator of immune responses. Existence of the closely related enzyme IDO2 was not discovered until two decades later (244, 245), and its physiological relevance is still to be fully elucidated (246, 247). Although it is expressed in a number of human tissues (245), IDO2 displays only low tryptophan-metabolizing activity (248) and frequently suffers from genetic polymorphisms compromising or ablating its activity (245). Nonetheless, studies performed with IDO2-deficient mice have indicated a role for the enzyme in controlling inflammation (249), and it has recently been associated with post-acute sequelae of SARS-CoV-2 (or long COVID-19 syndrome) (250). Finally, expression of TDO is mainly restricted to the liver and the brain, where it constitutively regulates systemic and brain tryptophan homeostasis (251, 252). In addition, TDO has been implicated in the maintenance of brain morphology and the regulation of brain function (252).

**Figure 10:**
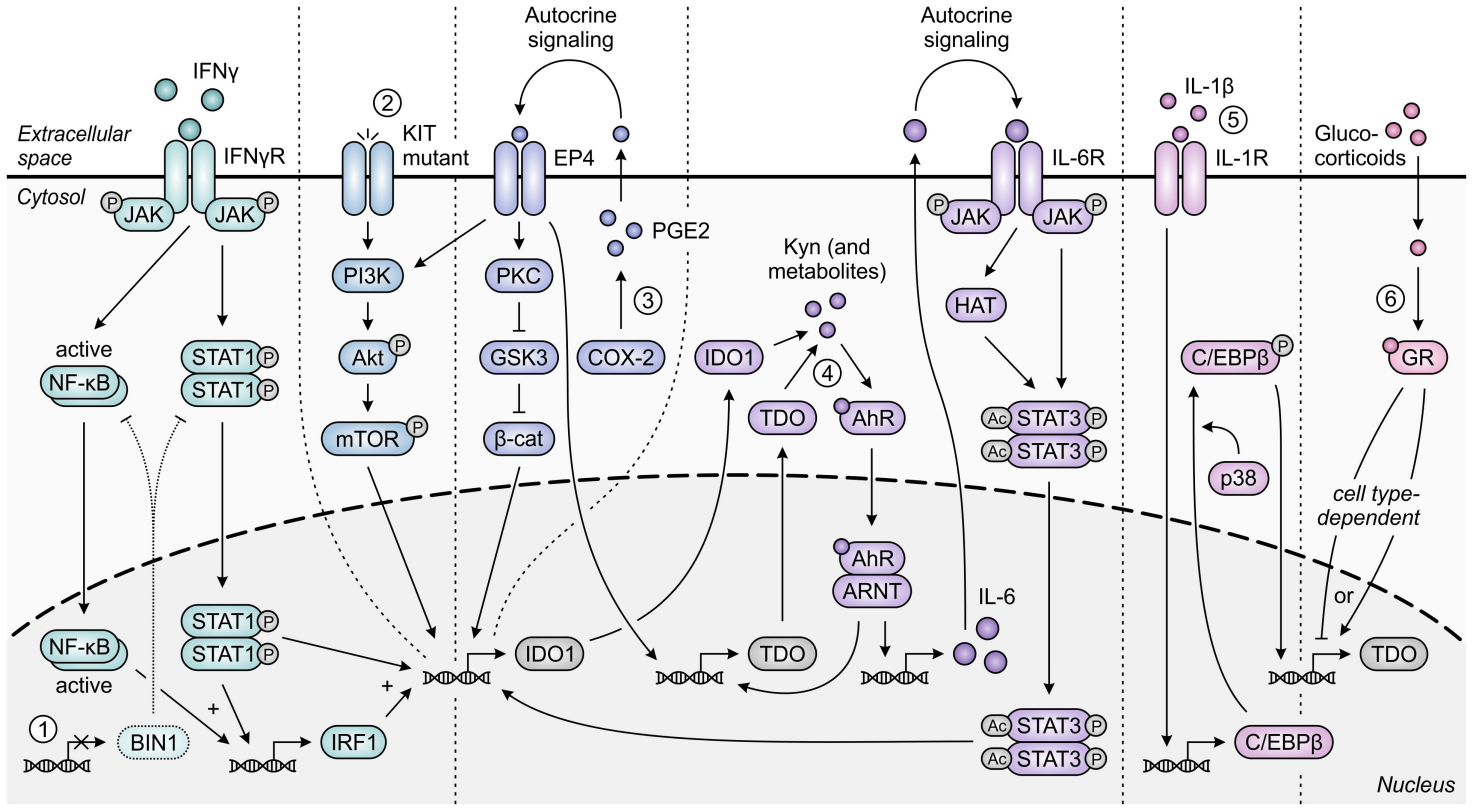
Regulation of IDO1 and TDO expression in tumor cells. IFNγ-induced expression of IDO1 is STAT1- and NF-κB-dependently increased upon loss of the tumor suppressor protein BIN1 [1]. Constitutive expression of IDO1 can additionally be enhanced through constitutive activation of the oncogenic KIT-PI3K-Akt-mTOR pathway upon *KIT* gain-of-function mutation [2]. Moreover, constitutive expression of both IDO1 and TDO is regulated through autocrine signaling involving the COX-2/PGE2 pathway [3] and through a positive feedback loop in which IDO1- and TDO-generated metabolites such as kynurenine (Kyn) promote expression of IDO1 and TDO through the AhR pathway [4]. For IDO1, the former pathway has been demonstrated to involve either KIT-PI3K-Akt-mTOR signaling or the PKC-dependent regulation of GSK3 and β-catenin (β-cat) activity, while the latter pathway involves autocrine IL-6/JAK/STAT3 signaling. Expression of TDO in tumor cells can also be C/EBPβ- and p38-dependently induced by IL-1β [5], and can be tumor cell type-dependently increased or decreased through glucocorticoid signaling [6]. Only pathway components with a demonstrated involvement in the different regulatory pathways are shown in the figure.

The immunoregulatory function of IDO1 is one also commonly exploited by tumors, and has additionally been ascribed to TDO expressed in the context of cancer. While normally silenced in many tissues, IDO1 is highly expressed in the tumor cells of a wide range of human cancer types (33). This constitutive or inducible expression can be initiated by loss of the tumor suppressor protein BIN1 (253), or gain-of-function mutation of the *KIT* proto-oncogene (**Figure 10**) (254). Moreover, constitutive IDO1 expression can further be maintained by autocrine signaling involving the cyclooxygenase-2 (COX-2)/prostaglandin E2 (PGE2) pathway (255) or a self-sustaining autocrine loop involving activation of the AhR by tryptophan metabolites (**Figure 10**) (256). Besides its presence in tumor cells, IDO1 is also expressed by various cells residing in the TME or tumor-draining lymph nodes, including dendritic cells, MDSCs, macrophages, endothelial cells, fibroblasts and mesenchymal stem cells (257–262). Across different tumor types, expression of IDO1 is inversely correlated with infiltrating CD3^+^ and CD8^+^ T cells as well as NK cells, while being positively correlated with regulatory T-cell frequency (263–268). In addition, elevated IDO1 expression correlates with tumor progression as well as poor survival in both solid and hematological cancers (269).

Expression of TDO can be detected in cancers of various tissue origins as well (35, 36), despite normally being confined to only specific tissue types. Constitutive expression of TDO in tumor cells can be regulated similarly to that of IDO1, involving signaling via the COX-2/PGE2 and AhR pathways (270, 271), while other TDO-regulating pathways have been described as well (**Figure 10**) (272, 273). Upregulated expression of TDO is additionally (or even predominantly) found in the stroma of various tumor types (274, 275), which includes its expression by pericytes and fibroblasts (274, 276). Elevated TDO expression is correlated with decreased survival in a number of human cancer types (277–280), and inversely correlates with CD8^+^ T-cell infiltration in human glioma tissues (36). In contrast to both IDO1 and TDO, gene expression of IDO2 is limited in human tumor tissues (37), and tryptophan-metabolizing activity fully resides with co-present IDO1 in IDO2-expressing tumors (281). Furthermore, although IDO2 is also expressed by dendritic cells (245), it remains unclear what role it serves in these cells (282, 283).

**Figure 11:**
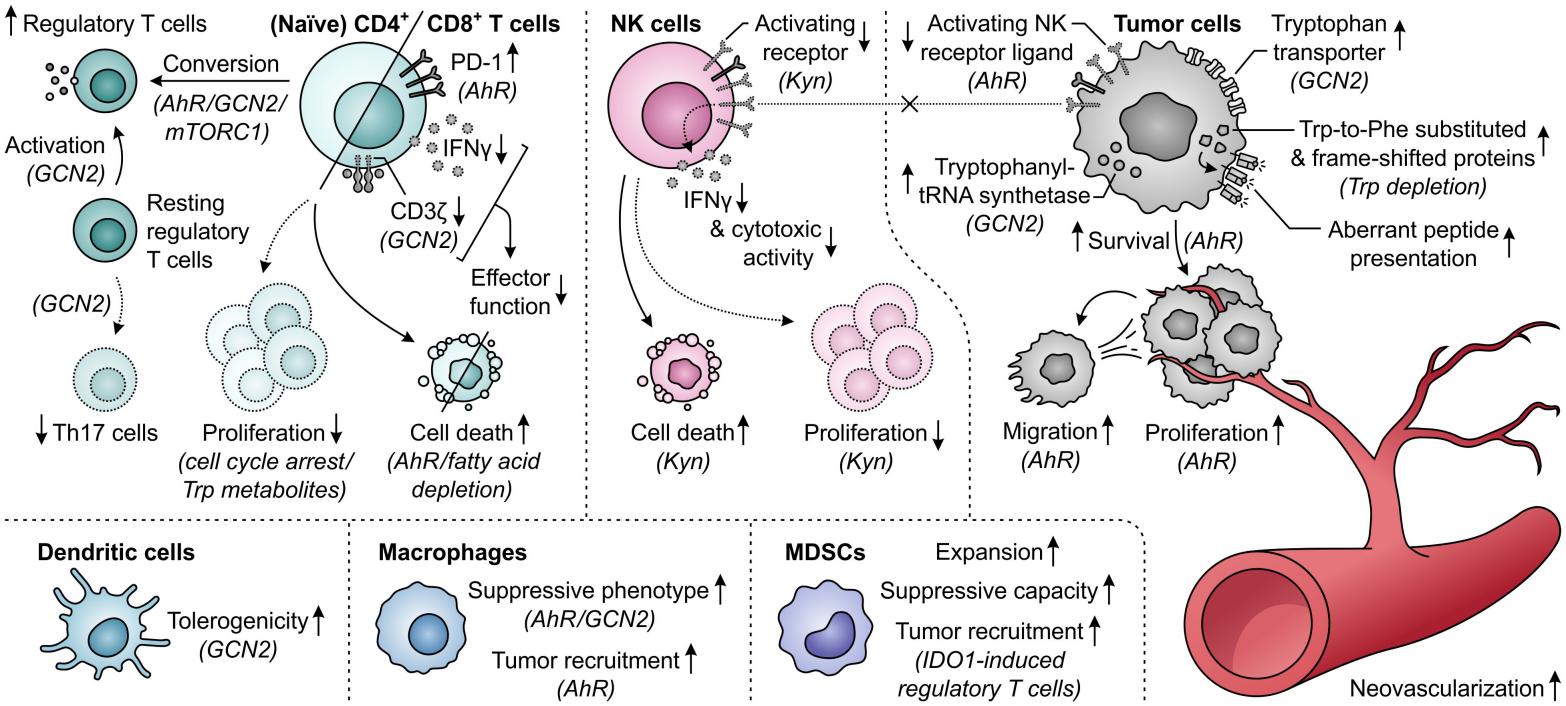
Effects of elevated tryptophan (Trp) metabolism by IDO1 and/or TDO on different immune cells, tumor cells and neovascularization. Molecular pathways, effector molecules or cells that are implicated in these effects are indicated within brackets. The molecular pathways include the general control nonderepressible 2 (GCN2) signaling pathway activated by Trp depletion, the mammalian target of rapamycin complex 1 (mTORC1) signaling pathway inhibited by Trp depletion, and the aryl hydrocarbon receptor (AhR) signaling pathway activated by kynurenine (Kyn) and downstream metabolites, as illustrated in **Figure 2**. Dotted outlines and arrows indicate components and processes which are downregulated or inhibited in response to elevated Trp metabolism.

Elevated metabolism of tryptophan by either IDO1 or TDO can adversely affect T-cell responses through both depletion of tryptophan levels and accumulation of its metabolites (**Figure 11**). While tryptophan depletion may act by activation of the GCN2 kinase pathway or repression of mTORC1 activity (284, 285), kynurenine and its downstream derivatives can operate as agonists of the AhR (**Figure 2**) (36, 44, 286, 287). Kynurenine uptake by T cells may additionally be potentiated by the depletion of tryptophan, as kynurenine and tryptophan are competitively transported into T cells by the SLC7A5/LAT1 transporter (288). IDO1-expressing cells inhibit the proliferation of activated T cells (257, 289, 290) and induce CD8^+^ T-cell anergy (284), which is at least partially ascribed to tryptophan depletion-induced GCN2 activation (284). Underlying molecular mechanisms affecting the T cells include their arrest in the mid-G_1_ cell cycle phase (289) and the GCN2-dependent downregulation of CD3ζ expression in the CD8^+^ subset, which impairs their cytotoxic effector function (**Figure 11**) (291). Notably, tryptophan metabolites may also contribute to suppression of T-cell proliferation as well as induce T-cell death (292–294), with AhR activation and potentially consequently enhanced fatty acid degradation as recently proposed cell death-inducing mechanisms (278, 295). Furthermore, elevated tryptophan metabolism upregulates PD-1 expression on CD8^+^ T cells through activation of the kynurenine–AhR signaling pathway (296–298), and concurrently inhibits the production of IFNγ by these cells (291, 299). On the other hand, IDO1-expressing cells promote the activity of immunosuppressive T cells through GCN2-dependent activation of resting regulatory T cells and inhibition of their reprogramming into Th17-like effector cells (300–302). Moreover, tryptophan depletion and metabolite accumulation promote the conversion of naïve CD4^+^ T cells into CD4^+^CD25^+^FoxP3^+^ regulatory T cells, which can involve modulation of either the GCN2, mTORC1 or AhR signaling pathway (**Figure 11**) (44, 147, 291).

The tryptophan-metabolizing activities of IDO1 and TDO can also have detrimental consequences for NK cells, which are mostly attributed to the action of kynurenine or its downstream metabolites (**Figure 11**). Effects exerted by these molecules include the inhibition of NK-cell proliferation and the induction of NK-cell death (293, 294). Moreover, kynurenine downregulates the expression of activating NK-cell receptors (303), as well as NK receptor ligand on tumor cells (304), with consequential inhibition of NK-cell cytotoxic activity and IFNγ production (303, 305). The generation of an immunosuppressive microenvironment can further be driven by the effects of excessive tryptophan metabolism on dendritic cells, macrophages and MDSCs (**Figure 11**). In particular, through modulation of the GCN2 pathway, tryptophan depletion enhances the tolerogenicity of dendritic cells (306) and polarizes macrophages towards a suppressive phenotype (307). The latter effect can also be induced through kynurenine-dependent AhR signaling (297, 308), which additionally promotes chemokine-mediated recruitment of these macrophages by tumors (308). Furthermore, IDO1 promotes the expansion and suppressive capacity of MDSCs (309), and indirectly recruits them to the tumor through the action of IDO1-induced regulatory T cells (**Figure 11**) (268).

In contrast to different immune cells, tumor cells can rather efficiently adapt to tryptophan-deprived conditions (**Figure 11**). While activation of the GCN2 pathway in malignant cells downregulates protein synthesis, it concurrently upregulates tryptophan transport (310) and tryptophanyl-tRNA synthetase expression (311), allowing the cells to readily utilize available tryptophan for protein synthesis to support proliferation. Notably, even upon sustained tryptophan deprivation, tumor cells continue protein synthesis, which was recently shown to result in the generation of frame-shifted and tryptophan-to-phenylalanine-substituted proteins (312, 313). Consequences of these aberrant proteins for cellular physiology are, however, still unclear and tumor cell presentation of resultant aberrant peptides may even elicit immunogenic responses (312, 313). Tryptophan metabolism can also directly promote the survival and proliferation of tumor cells through accumulation of kynurenine (36, 314, 315), which additionally stimulates their migratory ability (270, 316). Moreover, the activity of IDO1 is involved in promoting neovascularization, thereby further supporting tumor growth (309, 317), while expression of TDO by tumor-associated pericytes suggests a similar role for TDO (**Figure 11**) (274).

Despite the abundant *in vitro* evidence indicating a tumor-promoting role for IDO1, elucidation of the effects exerted by IDO1 *in vivo* have been complicated by the expression of IDO1 in both tumor cells and non-malignant host cells of cancer patients, of which contributions vary among cancer types (275). This encumbers the reproduction of tumors and their TME in syngeneic mouse models, which is further obstructed by the scarcity of murine cell lines naturally expressing IDO1 (318), as is also the case for TDO (319). However, using IDO1-deficient mice, host IDO1 has been found to play a role in promoting tumor growth, MDSC accumulation and expression of PD-1 by CD8^+^ T cells in a model of Lewis lung carcinoma (320). In murine models of colon, skin and brain cancer, however, host IDO1 deficiency does not diminish tumor growth (321–323), although it modestly enhances the efficacy of immune checkpoint blockade therapies (322) or decreases regulatory T-cell infiltration (323). Notably, ablation of host IDO1 expression also induces loss of IDO1 inhibitor efficacy in different tumor models, which would be suggestive of a suppressive role for host IDO1, although deficiency in host IDO1 itself paradoxically does not alter tumor growth in these models (324, 325). On the other hand, tumor cell-specific knockdown of IDO1 can inhibit tumor growth, decrease regulatory T-cell accumulation and improve survival in murine cancer models (321, 326). An important role for tumor-expressed IDO1 is further corroborated by the effective IDO1 inhibitor-mediated suppression of tumor growth in IDO1-deficient hosts (327). Moreover, IDO1 overexpression in tumor cells has been shown to promote tumor growth *in vivo* (33, 268), which is associated with decreased infiltration of effector T cells, and increased numbers of regulatory T cells and MDSCs (268). Based on *in vivo* models, tumor cell-expressed IDO1 thus appears to considerably contribute to the promotion of tumor growth, whereas a role for host IDO1 remains to be further substantiated.

Since discovery of the immunosuppressive role of IDO1 in tumor development, a great number of IDO1 inhibitors has been developed (328, 329). Preclinical evaluation of promising inhibitors has demonstrated their lymphocyte-dependent monotherapeutic efficacy in different murine tumor models (268, 299, 325, 327, 330). Moreover, IDO1 inhibition enhances the *in vivo* efficacy of α-CTLA-4 and α-PD-(L)1 therapy (322, 331–333), which may in part be related to the induction of IDO1 observed upon immune checkpoint blockade (330, 331). Similar induction of IDO1 also appears to occur in patients, as indicated by the elevation of systemic kynurenine-to-tryptophan ratios in α-PD-1-treated sarcoma, melanoma and renal cell carcinoma patients (334, 335). Contrary to inhibitors of IDO1, only relatively few selective TDO inhibitors have been reported (336–339). Development of TDO inhibitors is complicated by the small size and lipophilicity of the TDO active site (337), explaining the general suffering of TDO inhibitors from limited potency or drug-likeness (339). Nonetheless, inhibitors such as the orally bioavailable LM10 and PF06845102/EOS200809 have enabled evaluation of the immunosuppressive effect of TDO in murine tumor models (35, 319). While TDO overexpression in tumor cells can promote tumor growth through suppression of the anti-tumor immune response (35, 36), inhibition of TDO can restore tumor suppression (35) and enhance the efficacy of CTLA-4 blockade therapy (319). Preclinical and early clinical development of several dual IDO1/TDO inhibitors is currently also ongoing (340–342), which may serve a role for tumors co-expressing IDO1 and TDO or those upregulating TDO upon IDO1 inhibition.

While clinical evaluation of selective TDO inhibitors for cancer treatment is still to be awaited, various IDO1 inhibitors have already entered clinical development, frequently in combination with immune checkpoint blockade (329). Epacadostat (INCB024360) was the first IDO1 inhibitor to advance into a phase III clinical trial (ECHO-301/KEYNOTE-252) after showing promising efficacy in advanced melanoma patients when combined with α-PD-1 antibodies in two nonrandomized, uncontrolled phase II trials (343, 344). However, the phase III trial failed to demonstrate an improved progression-free survival for the combination of epacadostat with pembrolizumab (α-PD-1) compared to pembrolizumab alone (345). Thereupon, several other phase III trials were terminated early, withdrawn or downscaled to randomized phase II trials, along with the suspension of several phase I and II evaluations (346). Nonetheless, a number of phase I and II trials has also been initiated since the failure of ECHO-301, suggesting that IDO1 inhibition may still have a future as an immunotherapeutic approach for cancer treatment.

**Figure 12:**
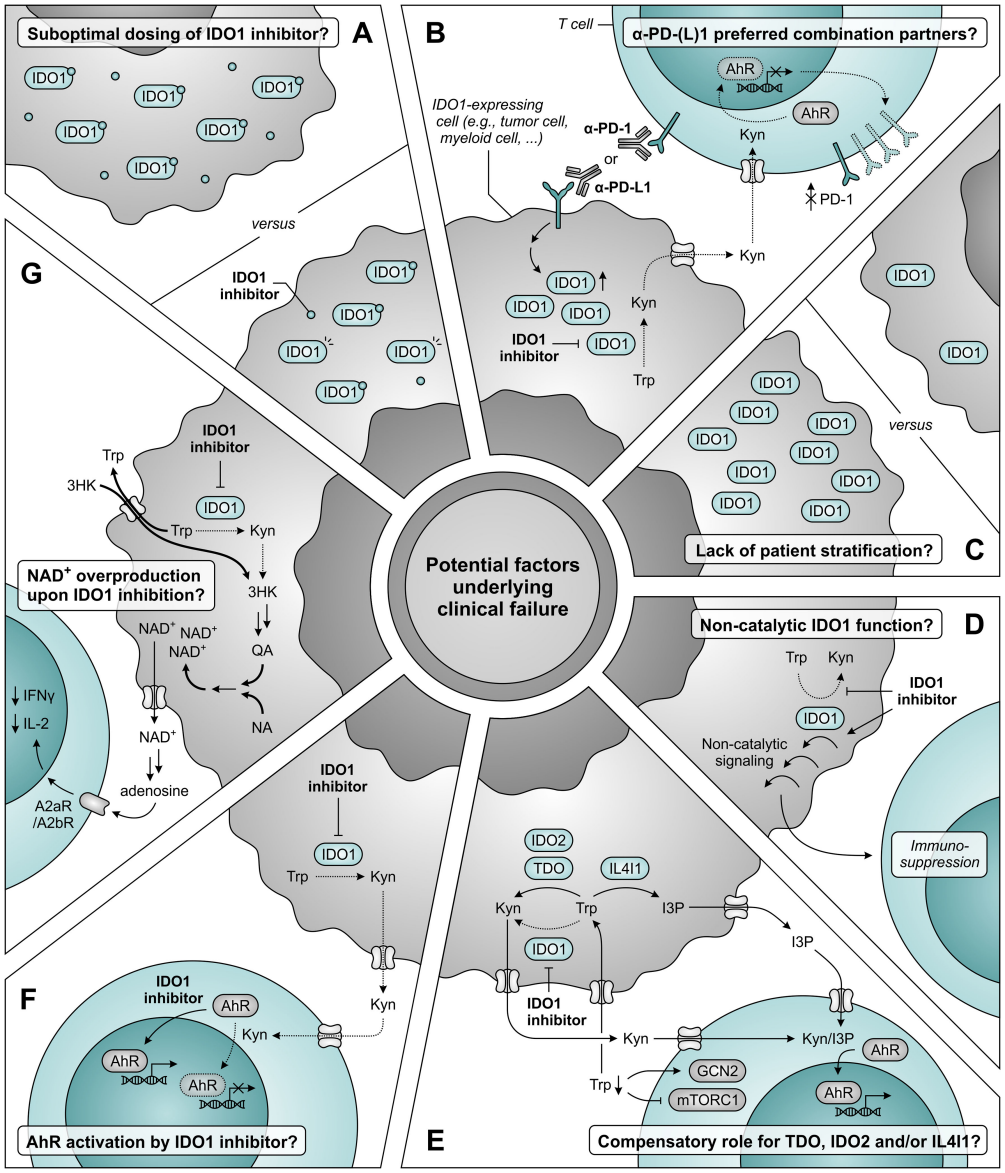
Potential factors underlying clinical failure of IDO1 inhibitor treatment. **(A)** The dosing of IDO1 inhibitor may be insufficient to obtain adequate intratumoral and intracellular concentrations required for maximal IDO1 inhibition. **(B)** α-PD-1 and α-PD-L1 antibodies may not be the ideal combination partners for IDO1 inhibitors based on the induction of IDO1 expression upon treatment with these antibodies (left), and the aryl hydrocarbon receptor (AhR)-dependent reduction of PD-1 expression on tumor-infiltrating T cells upon IDO1 inhibition (right). **(C)** A lack of patient stratification based on expression or activity of IDO1 may also underly the absence of clinical efficacy. **(D)** IDO1 may induce immunosuppression through a non-catalytic function that is not restrained (and may even be enhanced) by inhibitors targeting the IDO1 active site. **(E)** Compensatory tryptophan (Trp) metabolism by either TDO, IDO2 and/or IL4I1 may negate the effects of IDO1 inhibition, either due to reduced competition for substrate or as a consequence of enzyme upregulation. IL4I1 catalyzes a different reaction compared to the other enzymes, but can also produce agonists of the AhR such as indole-3-pyruvic acid (I3P). **(F)** IDO1 inhibitors may act as AhR agonists themselves, thereby nullifying the reduced activation of AhR by kynurenine (Kyn). **(G)** Inhibition of IDO1 may induce nicotinamide adenine dinucleotide (NAD^+^) overproduction through induction of transporter and metabolic enzyme expression, with consequential suppression of T-cell proliferation and function. Dotted arrows and outlines indicate processes which are inhibited or downregulated, whereas bold arrows indicate upregulated processes.

Extensive discussion about factors underlying the disappointing clinical results has also since arisen (**Figure 12**) (347–349). Concerns have been raised as to whether the dosing of epacadostat in the ECHO-301 trial was sufficient to obtain adequate intratumoral and intracellular concentrations required for maximal IDO1 inhibition (**Figure 12A**) (347). Based on results of a phase I dose escalation, the chosen dose of 100 mg twice daily yields an appreciable, though sub-maximal, reduction in plasma kynurenine levels (350). Considering the reported association of immune checkpoint blockade with induction of IDO1 (330, 331, 334, 335), dosing based on its monotherapeutic profile could thus have proven to be insufficient. Regrettably, the degree of IDO1 inhibition was evaluated in neither plasma nor tumor biopsies of patients enrolled in the ECHO-301 trial. The same argument of therapy-induced IDO1 induction should also question α-PD-1 antibodies as preferred combination partners for IDO1 inhibitors. Moreover, restriction of kynurenine-mediated PD-1 induction in tumor-infiltrating T cells upon IDO1 inhibition may even cause PD-1 blockade to be redundant (**Figure 12B**) (296–298). Stratification of patients based on IDO1 expression or activity could also have improved the chance of observing a clinical benefit upon IDO1 inhibition, but this is still only rarely applied in clinical trials (**Figure 12C**) (347, 349, 351).

IDO1 can also exert immunosuppressive effects independent of its enzymatic activity (352–354). This involves non-catalytic signaling events initiated by the enzyme (352, 353), which are likely not inhibited by active site-targeting inhibitors, and may even be enhanced by them (355) (**Figure 12D**). Such effects were recently suggested to partially account for the IDO1-dependent immune suppression observed in human glioma (354) and were demonstrated to promote tumor growth in a melanoma mouse model (356), but it remains to be determined whether they also serve a role in different tumor types. Other possible explanations for the outcome of the ECHO-301 trial include a potential compensatory role of TDO, IDO2 and/or IL4I1 (**Figure 12E**) (37, 348), and activation of AhR signaling by IDO1 inhibitors themselves (**Figure 12F**) (349, 357). Although these mechanisms are unlikely to account for the disappointing results (349), only thorough evaluation of clinical samples will allow their unequivocal rejection. Finally, a recent study performed with treatment-naïve ovarian cancer patients demonstrates that IDO1 inhibition by epacadostat induces overproduction of tumoral nicotinamide adenine dinucleotide (NAD^+^), which reduces T-cell proliferation and functionality *in vitro* and mitigates IDO1 inhibitor efficacy *in vivo* (**Figure 12G**) (358). Blockade of NAD^+^ generation or signaling may therefore present a promising combination strategy for IDO1 inhibition (358). Alternatively, patients may benefit from the direct targeting of downstream effector pathways of tryptophan metabolism, such as AhR or GCN2 signaling (297, 359), or the selective depletion of kynurenine by kynureninase treatment (360), rather than inhibition of upstream IDO1 or TDO. Moreover, peptide vaccines are being developed and clinically tested to target IDO1-expressing cells rather than the enzyme itself (361), and degraders are in development to target both enzymatic and non-enzymatic IDO1 activities (362). However, these strategies remain to be further validated in preclinical or clinical studies.

Collectively, the extensive prognostic, preclinical and early-phase clinical evidence linking deranged tryptophan metabolism to immunosuppression and tumor growth once positioned IDO1 inhibitors at the forefront of experimental immunotherapy. While this perception has since been challenged by the failure of the ECHO-301 trial, it remains evident that tryptophan metabolism holds promise as a targetable pathway for cancer treatment. Nonetheless, this development urges for a deeper understanding to be attained of both the mechanisms underlying IDO1-mediated immunosuppression and the effects of tryptophan metabolic pathway inhibition, to which the examination of clinical samples may strongly contribute. Moreover, the improved design of clinical trials using patient stratification and monitoring of target engagement could contribute to further validation of the target, whereas alternative or combinatorial strategies should also still offer hope for cancer patients.

## 7 The aromatic amino acid-metabolizing enzyme IL4I1

The final amino acid-metabolizing enzyme with a proposed role in tumor immune escape is IL4I1, which is a secreted l-amino acid oxidase (LAAO) first characterized by its interleukin 4-inducible expression in murine and human B cells (363, 364). IL4I1 catalyzes the conversion of l-amino acids into their respective α-keto acids with concomitant release of hydrogen peroxide (H_2_O_2_) and ammonia (NH_3_), and has a preference for aromatic substrates, specifically phenylalanine, tyrosine and tryptophan (**Figure 1E**) (37, 365–367). Although five isoforms of IL4I1 are encoded by the human genome, expression of only two isoforms has been found in humans (363, 368). These isoforms diverge in their secretory signal peptide sequence, but yield identical proteins upon signal peptide cleavage (368). Expression of the first IL4I1 isoform is chiefly found in lymphoid tissues (364, 369), while the second isoform is highly expressed in the testis and can also be found in specific cells of the brain (368). Within human lymphoid tissues, IL4I1 expression is primarily restricted to professional antigen-presenting cells (364, 366, 370), with considerably higher levels found in macrophages and dendritic cells compared to those in B cells (371). Moreover, IL4I1 is expressed by human Th17 and Th17-like cells (372–374) and is found in MDSCs of tumor-bearing mice (31, 375). While the physiological roles of IL4I1 remain to be fully elucidated, the enzyme has been ascribed various immunoregulatory functions, including regulation of B-cell physiology (376).

Elevated expression of IL4I1 has been detected in various human cancer types, including both solid tumors and lymphomas (37, 369, 370). In melanoma and ovarian cancer patients, IL4I1 expression or activity is additionally found to increase with disease progression or metastasis (37, 377, 378). Among solid and non-B-cell malignancies, IL4I1 is only rarely expressed by tumor cells, but instead can often be found in tumor-associated macrophages (370, 379). Contrarily, although IL4I1 expression by macrophages is a common feature of B-cell lymphomas as well, several subtypes also frequently express IL4I1 in neoplastic cells, in keeping with its natural expression in B cells (370). Moreover, whereas high IL4I1 expression appears to portend a poor prognosis in several solid tumor types (37, 378, 380–383), high levels of IL4I1 are correlated with superior outcome in follicular and diffuse large B-cell lymphoma (370, 384). This suggests a dichotomous role for IL4I1 in solid tumors compared to B-cell lymphomas, which may be related to the physiological regulatory role of IL4I1 in B-cell activation (376), but requires further substantiating evidence.

**Figure 13:**
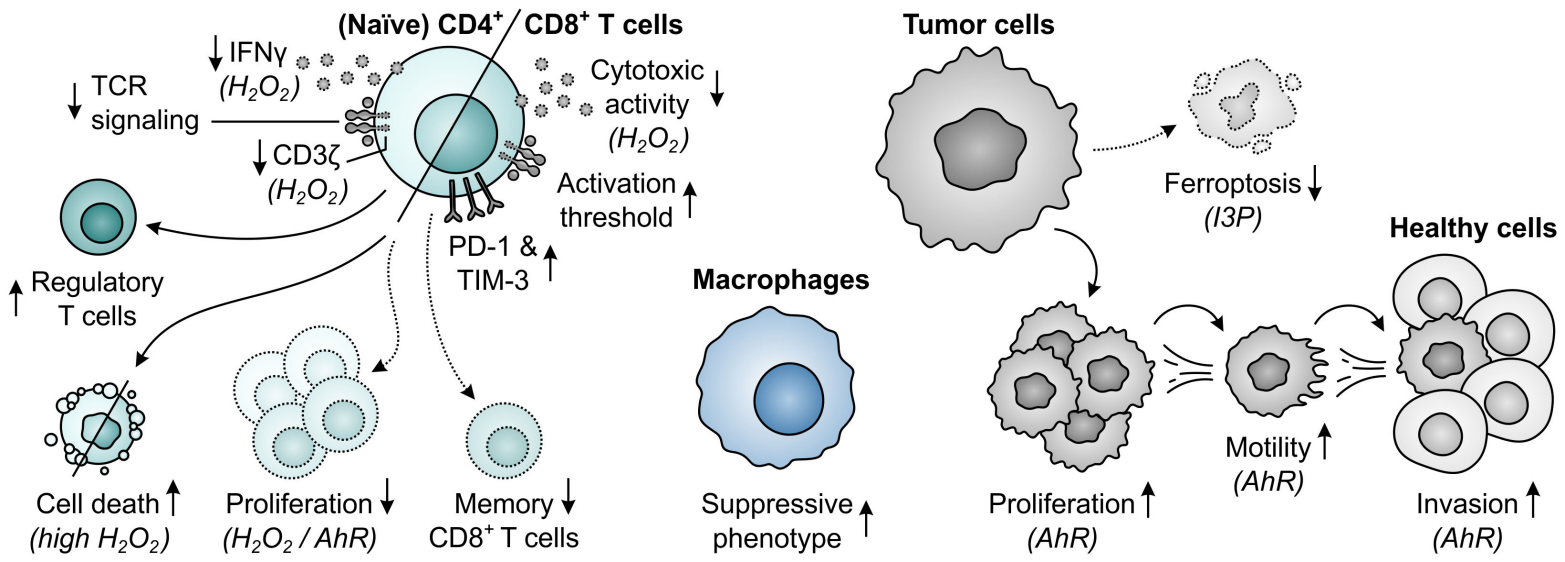
Effects of elevated phenylalanine, tyrosine or tryptophan metabolism by IL4I1 on T cells, macrophages and tumor cells. Molecular pathways and effector molecules that are implicated in these effects, such as activation of the aryl hydrocarbon receptor (AhR) by indole-3-pyruvic acid (I3P) or other tryptophan metabolites, are indicated within brackets. Dotted outlines and arrows indicate components and processes which are downregulated or inhibited in response to elevated metabolism by IL4I1.

Similar to other amino acid-metabolizing enzymes, IL4I1 is capable of inhibiting the proliferation of activated human T cells (**Figure 13**) (366). This effect was initially solely ascribed to the IL4I1-mediated generation of H_2_O_2_, since phenylpyruvic acid, the α-keto acid product of major substrate phenylalanine, only affects T cells at very high concentrations (366). Treatment of human T cells with H_2_O_2_ results in the downmodulation of CD3ζ expression, IFNγ production and cytotoxic activity (366, 385, 386). Moreover, at higher concentrations or prolonged exposure, H_2_O_2_ can induce T-cell death (387, 388), which preferentially targets effector rather than regulatory T cells (389). IL4I1 activity additionally promotes the differentiation of naïve CD4^+^ T cells into CD4^+^CD25^+^FoxP3^+^ regulatory T cells, which has been suggested to involve phenylalanine deficiency-induced inhibition of mTORC1 signaling (390). Based on the relatively low affinity of IL4I1 for phenylalanine (391), however, it seems unlikely that IL4I1 is capable of depleting this amino acid, indicating that another mechanism may underly the observed effect. Finally, IL4I1 is capable of raising the activation threshold of CD8^+^ T cells while restraining their differentiation into memory T cells (392), and promoting the polarization of macrophages towards a suppressive phenotype (**Figure 13**) (393, 394).

More recently, IL4I1 has additionally been recognized for its ability to generate AhR ligands upon degradation of one of its other substrates (37). Metabolism of tryptophan yields the α-keto acid indole-3-pyruvic acid, which can subsequently be converted into indole-3-lactic acid, indole-3-acetic acid, indole-3-aldehyde and kynurenic acid (**Figure 1E**) (37, 395). Each of these metabolites has previously, albeit generally not consistently, been associated with AhR agonism (37, 286, 395–398), with indole-3-pyruvic acid serving as its most potent activator (37). Addition of indole-3-pyruvic acid, but not the α-keto acid derivative of phenylalanine or tyrosine, to human T cells induces the expression of AhR target genes, and reproduces the inhibition of their proliferation observed with supernatants of IL4I1-expressing cells (**Figure 13**) (37). IL4I1 is also reported to inhibit TCR signaling through a mechanism independent of its enzymatic activity (399), but an effect of IL4I1-mediated tryptophan metabolism was not considered in this study and may underly these observations. The activity of IL4I1 can additionally directly affect tumor cells, as evidenced by the enhanced proliferation, motility and invasive capacity of IL4I1-expressing tumor cells of different tissue origin (37, 383, 400), which may at least in part be due to indole-3-pyruvic acid-mediated activation of the AhR pathway (37). Similarly, AhR signaling may be involved in the IL4I1-induced suppression of tumor cell ferroptosis (**Figure 13**) (367).

Across human cancer types, IL4I1 gene expression associates more strongly with a pan-tissue AhR activation signature compared to that of either IDO1 or TDO, indicating IL4I1-generated metabolites as key AhR activators in cancer (37). While this is corroborated by the ability of IL4I1 to activate the AhR in various models and the high expression of IL4I1 in cancer tissues (37), it is argued by another study demonstrating only poor correlation between the expression of IL4I1 and individual AhR target genes in a number of tumor types (391). Moreover, IL4I1 demonstrates a roughly 50-fold lower affinity for tryptophan compared to IDO1 (391), whereas its expression is on average only moderately higher than that of IDO1 across different tumor types (37). Although this may partially be compensated for by the considerably higher AhR-activating potency of indole-3-pyruvic acid compared to IDO1-generated metabolites (37), it remains unclear whether IL4I1 is capable of achieving significant AhR activation in most tumor types. Nonetheless, metabolomics studies have demonstrated high levels of the phenylalanine- and tyrosine-derived metabolites phenylpyruvic acid and 4-hydroxyphenylpyruvic acid in ovarian cancer patient samples (377, 401). While there is currently no indication for a role of these metabolites in cancer development or immunosuppression, these findings suggest considerable activity of IL4I1 in these patients. Accordingly, when IL4I1 is relieved from competition for tryptophan upon inhibition of IDO1, IL4I1-mediated AhR activation could present a mechanism of resistance against IDO1 inhibition (37, 377). While this remains to be validated in specimens of patients treated with an IDO1 inhibitor, it may argue for the treatment of patients with a combination of IDO1 and IL4I1 inhibitors, or with an AhR inhibitor.

The *in vivo* effect of IL4I1 expression on tumor growth and the anti-tumor immune response has currently been studied in only a limited number of models. In melanoma-challenged, tumor antigen-immunized mice, IL4I1 expression by tumor cells facilitates tumor outgrowth, which is mediated by suppression of the tumor antigen-specific CD8^+^ T-cell response (402). Similarly, tumoral IL4I1 overexpression reduces CD8^+^ T cell infiltration into tumors of non-immunized melanoma-transplanted mice (403), although it does not affect tumor growth likely due to the poorly immunogenic, highly aggressive nature of this model (402, 403). In contrast, knock-out of IL4I1 in B cells reduces the growth of melanoma cells in transplanted mice, which is accompanied by increased proportions of effector memory T cells and reduced proportions of regulatory T cells and G-MDSCs in the tumor microenvironment (404). In a model of spontaneous melanoma, IL4I1 expression and activity increases with disease progression (404, 405), while IL4I1 deficiency enhances tumor control and associates with reduced G-MDSC and macrophage infiltration as well as enhanced CD4^+^ T-cell, CD8^+^ T-cell and B-cell infiltration into the tumor (405). Similarly, IL4I1-deficient mice challenged with chronic lymphocytic leukemia present with reduced tumor burden, which is accompanied by enhanced CD8^+^ T-cell functionality and reduced frequency of total and activated regulatory T cells (37). Finally, in a model of Lewis lung carcinoma, IL4I1 knock-out inhibits tumor growth and increases the proportions of CD8^+^ T cells and pro-inflammatory macrophages, while decreasing the proportion of regulatory T cells and immunosuppressive macrophages (406). Overall, these results corroborate the findings of enhanced MDSC and regulatory T-cell infiltration and reduced CD8^+^ T-cell infiltration in cancer patients with high IL4I1 expression (37, 378, 406).

Similar as observed for IDO1, α-PD-1 treatment can also induce IL4I1 upregulation in cancer patients, and IL4I1 is suggested to potentially constitute a resistance mechanism to immune checkpoint blockade (37). Moreover, IL4I1 induces expression of the inhibitory checkpoint proteins PD-1 and TIM3 on CD8^+^ T cells upon co-culture with patient-derived tumor organoids (**Figure 13**) (407), suggesting that IL4I1 inhibitors may need to be combined with immune checkpoint blockade for effective cancer treatment. Whereas only few inhibitors of IL4I1 have yet been disclosed (408–411), the preclinical inhibitor CB-668 has demonstrated both monotherapeutic efficacy and favorable combinatorial efficacy with α-PD-L1 therapy in tumor-bearing mice (412).

Taken together, although the field studying the role of IL4I1 in cancer immune escape is still in its relative infancy compared to that of IDO1, the pharmacological targeting of IL4I1 offers new prospects for the treatment of cancer patients. A more comprehensive understanding of the consequences of IL4I1 expression and its inhibition in cancer is, however, required to determine whether this enzyme indeed constitutes an effective immunotherapeutic target. As the development of IL4I1 inhibitors is currently ongoing, disclosure of complementary studies addressing these aspects may be expected in the near future.

## 8 Concluding remarks

Cancer immunotherapy has revolutionized the treatment of malignant tumors, with immune checkpoint blockade as major breakthrough therapy being approved for an ever-expanding list of clinical indications. However, despite the impressive durable responses observed in a subset of cancer patients, diverse resistance mechanisms limit the effectiveness of immunotherapeutic interventions. Amino acid-metabolizing enzymes can serve as important mediators of tumor immune evasion, as activated T cells and other immune cells have a high need for amino acids and can be adversely affected by accumulation of amino acid-derived metabolites. Inhibition of tumor- or immunosuppressive cell-expressed GLS1, ARG1, iNOS, IDO1, TDO and/or IL4I1 therefore constitutes a promising approach to alleviate tumor-induced immune suppression, either as monotherapy or in combination with other treatment modalities.

Clinical successes of inhibitors of amino acid-metabolizing enzymes are, however, still limited to date, indicating that our understanding of the involved complex pathways remains incomplete. This urges for both the extension of preclinical research as well as the thorough investigation of samples collected during clinical studies. As various clinical trials are currently also still ongoing, valuable insight into the efficacy of different inhibitors is also still to be expected. However, identification of novel targets such as IL4I1, exploration of alternative strategies such as targeting of downstream pathways (*e.g.*, the AhR pathway) and rational selection of combination therapies should additionally receive adequate focus, as these may well serve to overcome the current limitations of cancer immunotherapies.
